# Supplementary material for: Diagnostic validation of novel Borrelia antigens discovered by whole-proteome microarray: Advancing early detection and test of cure for Lyme disease
Source: Cell Rep Med. 2025 May 1;6(5):102097. doi: 10.1016/j.xcrm.2025.102097 (PMC12147846; doi:10.1016/j.xcrm.2025.102097)
Supplement: Document S1. Figures S1–S7 and Tables S1–S4 [file mmc1.pdf]

Cell Reports Medicine, Volume *6*

Supplemental information

Diagnostic validation of novel *Borrelia* antigens discovered by whole-proteome microarray: Advancing early detection and test of cure for Lyme disease

Abhijeet Nayak, M.E. Baarsma, Jacqueline A. van Eck, Arlo Z. Randall, Jeanine Ursinus, Andy A. Teng, Jozelyn V. Pablo, Chris Hung, Doris U.M. Wopereis, Freek van de Schoor, Calin D. Popa, Cees C. van den Wijngaard, Bart-Jan Kullberg, Leo A.B. Joosten, Herman Kuiper, Joseph J. Campo, Xiaouw Liang, and Joppe W. Hovius

Supplemental data

Figures

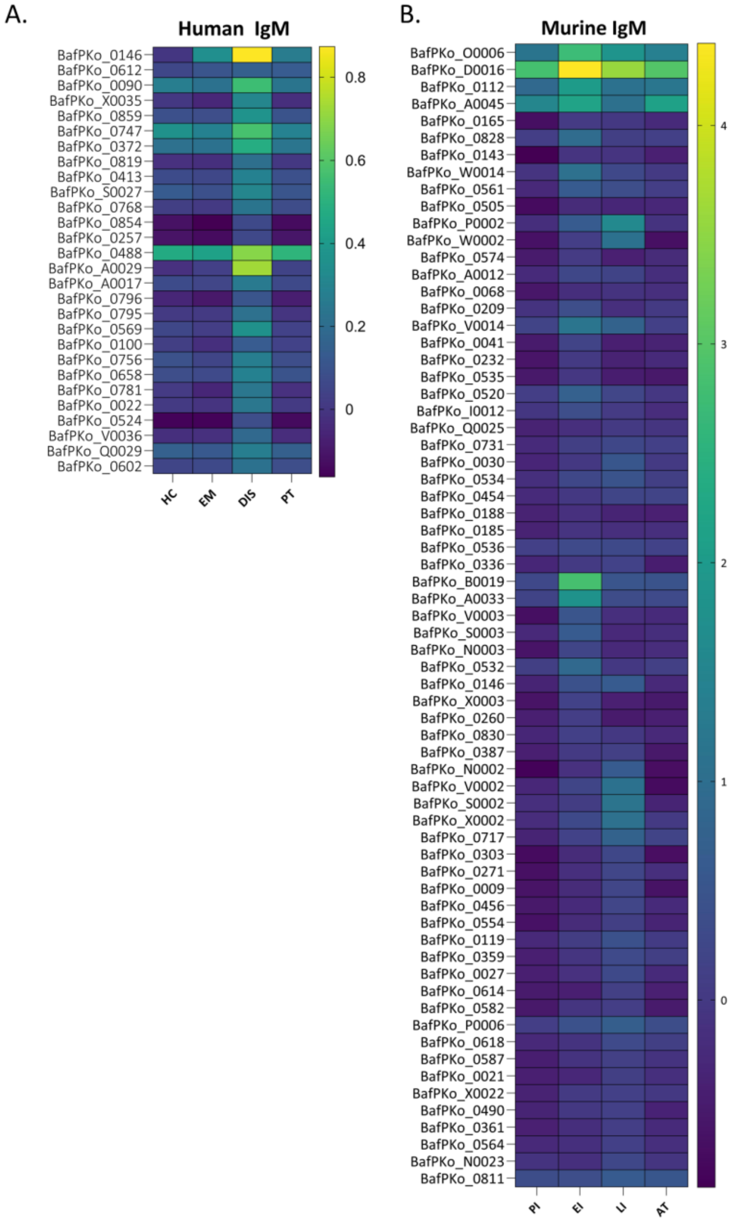

Supplementary figure S1. ***Comprehensive screening of B. afzelii proteome microarray with the discovery cohort and the murine cohort revealed several unique and known Borrelia antigens.*** Related to figure 1. 1296 *B. afzelii* proteins were printed on a microarray chip and probed with human sera from the discovery cohort (HC; n=50, EM; n=49, DIS; n=25, PT; n=25) and murine cohort (EI, PI, LI, AT; n=8) to identify immunoreactive antigens. **B. IgM antigens in the human discovery cohort**: Heat map depicting IgM reactivity of selected antigens in the discovery cohort at different stages of disease as compared to healthy controls. **C. IgM antigens in murine cohort**: Heat map depicting IgM selected murine antigens at different infection stages as compared to the pre-immune controls. Heat maps represent the mean of normalized signal intensity of each antigen per group for both human and murine cohorts. Heatmaps were created using GraphPad Prism v10.2.0. All antigens including the positive and negative controls were spotted in duplicates on the microarray slides. **DC**: Discovery cohort; **HC**: Healthy controls; **EM**: Erythema migrans; **DIS**: Disseminated disease; **PT**; Post-treated ; **PI**: Pre-immune; **EI**: Early infection; **LI**: Late infection and **AT**: Antibiotics treated.

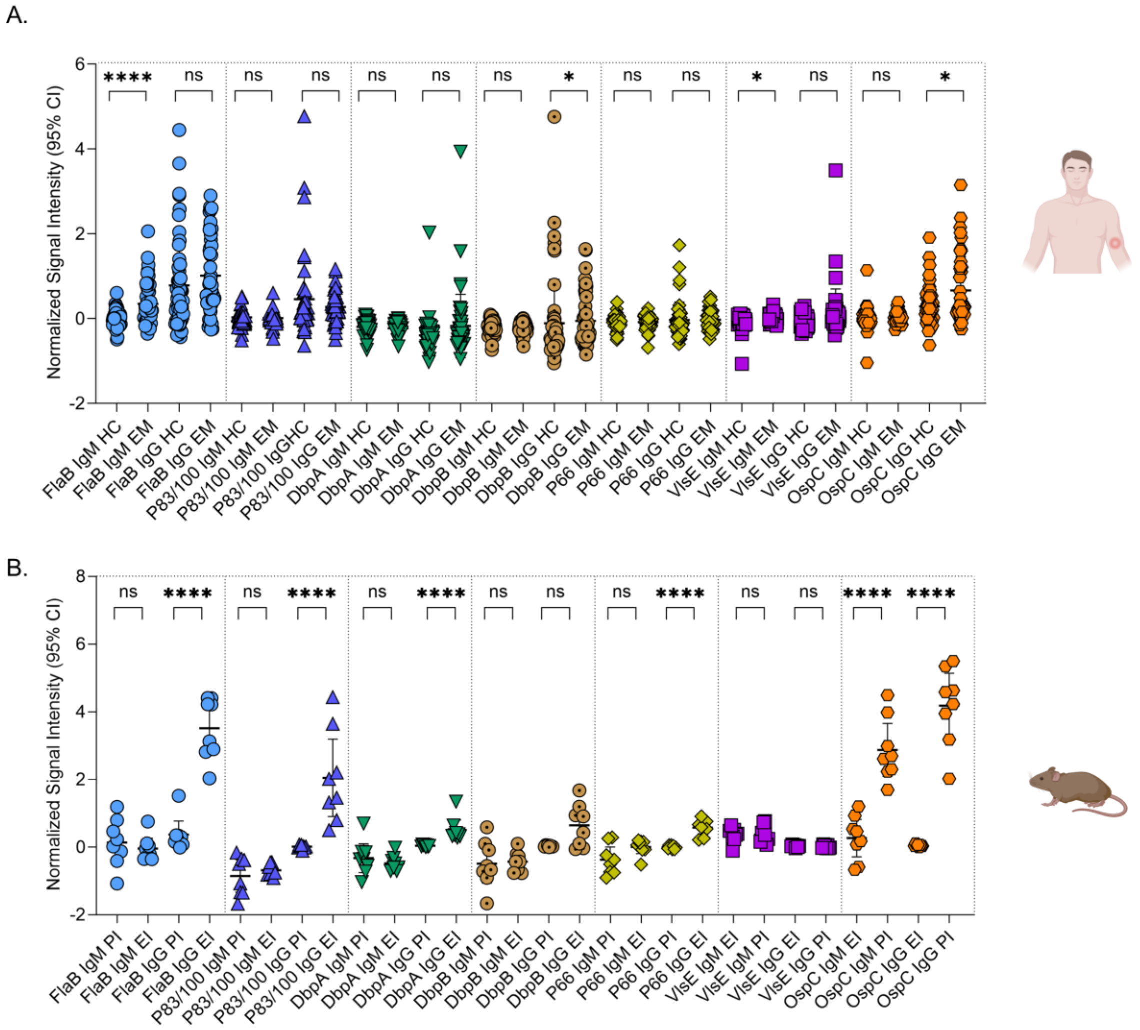

Supplementary figure S2. IgM and IgG responses of known *B. afzelii* antigens identified commonly in the discovery cohort and murine sera. Related to Figure 1. **A. IgM and IgG responses in the discovery cohort:** X-axis represents EM (n=49) and HC (n=50). Y-axis represents normalized signal intensity with error bars representing 95% CI. **C. IgM and IgG responses in murine cohort:** X-axis represents EI (n=8) and PI (n=8) stage. Y-axis represents mean normalized signal intensity with error bars representing 95% CI. Statistical significance was calculated using unpaired non-parametric Mann-Whitney test, ****p<0·0001, ***p<0·001, **p<0·01, *p<0·05, and ns- non-significant. All antigens including the positive and negative controls were spotted in duplicates on the microarray slides. Figures created using Biorender.com and GraphPad Prism v10.2.0. **HC**: Healthy controls; **EM**: Erythema migrans; **PI**: Pre-immune and **EI**: Early infection.

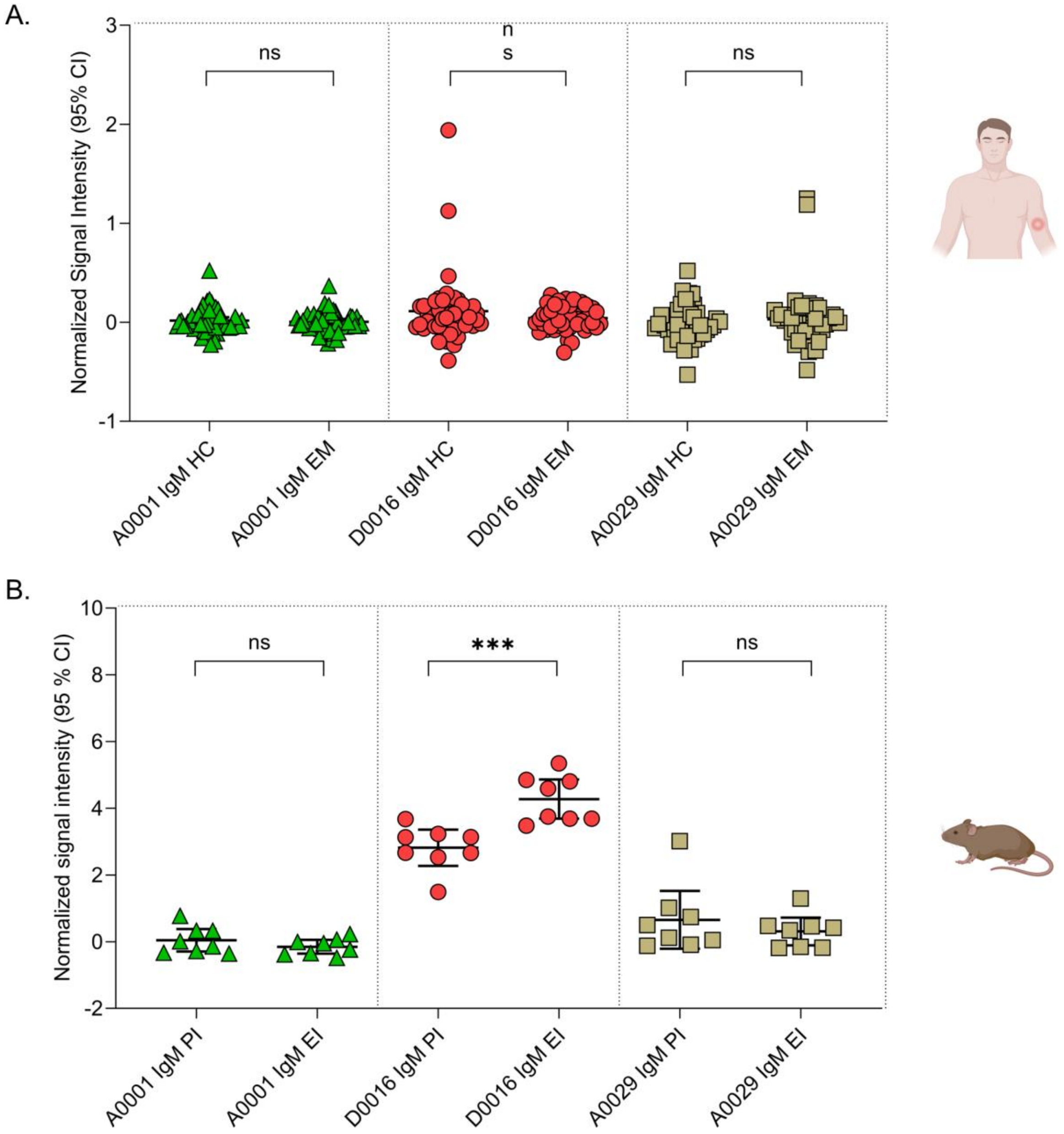

Supplementary figure S3. IgM responses. of A0001, D0016 and A0029: **A. Discovery cohort.** Related to Figure 2. X-axis represents EM sera (n=49) and HC sera (n=50) stage for A0001, D0016 and A0029, respectively. Y- axis represent normalized signal intensity with 95% CI. **B. Murine cohort:** X-axis represents EI sera (n=8) and PI sera (n=8) stage for A0001, D0016 and A0029, respectively. Y- axis represent normalized signal intensity with 95%CI. Figures were created using Biorender.com and GraphPad Prism v10.2.0 and statistical significance was calculated using unpaired non-parametric Mann-Whitney test in GraphPad Prism, ****p<0·0001, ***p<0·001, **p<0·01, and ns- non-significant. All antigens including the positive and negative controls were spotted in duplicates on the microarray slides. **HC**: Healthy controls; **EM**: Erythema migrans; **PI**: Pre-immune and **EI**: Early infection.

A. BafPKo_A0001

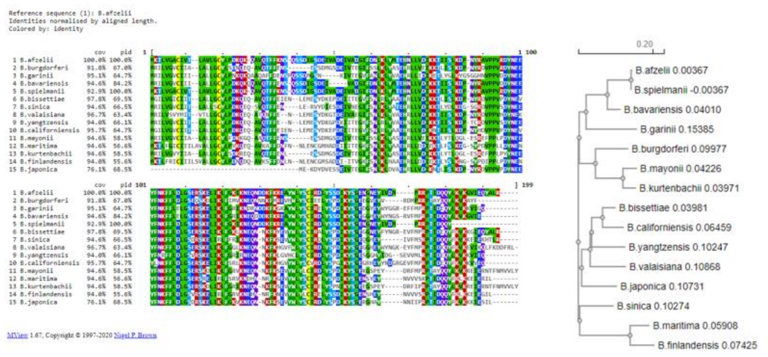

B. BafPKo_D0016

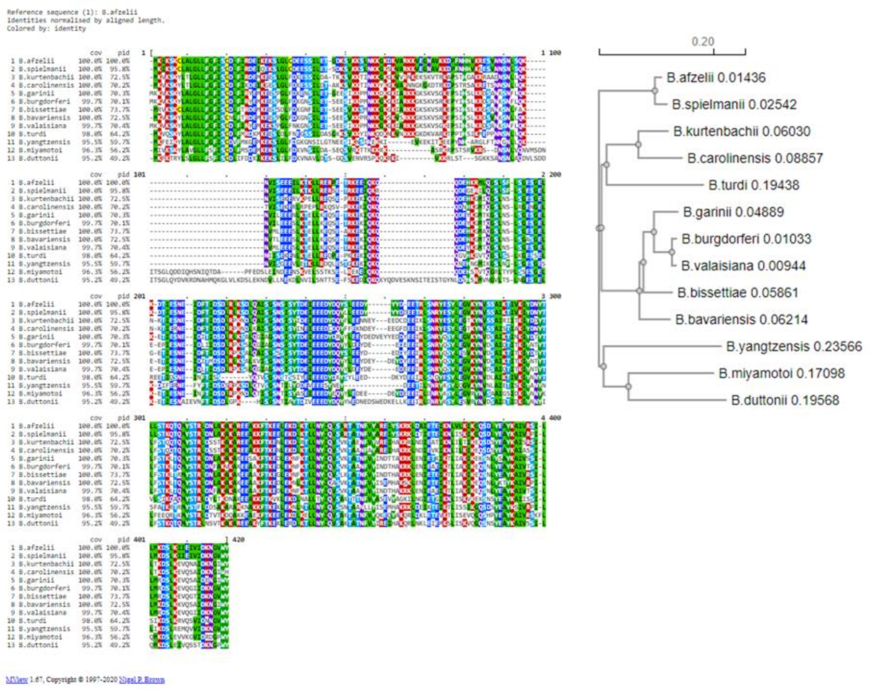

C. BafPKo_A0029

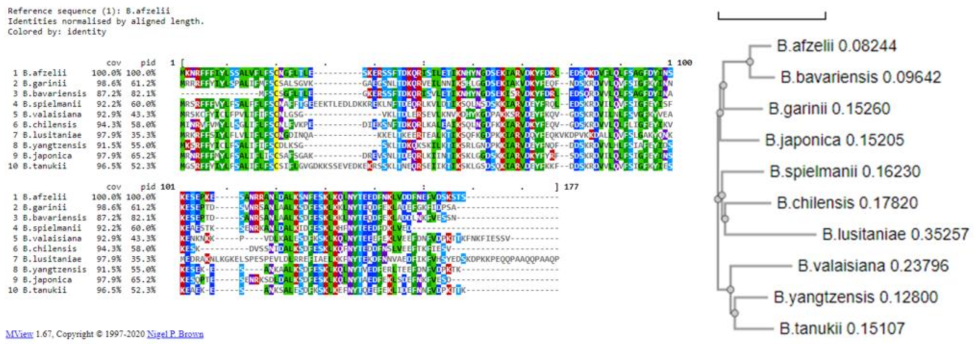

Supplementary figure S4. Sequence alignment and percent identity matrix of novel *B. afzelii* antigens. A. A0001, B. D0016 and C. A0029. Related to Figure 2. Sequence alignment files were created using Clustal Omega EMBL-EBI and alignments were created with MView Multiple sequence alignment tool EMBL-EBI. NCBI BLAST was performed, and all displayed sequences were included in the analysis. For multiple annotations from the same spirochetal genospecies only the sequence with the highest E-value was included. Phylogenetic trees were generated using Clustal Omega EMBL-EBI.

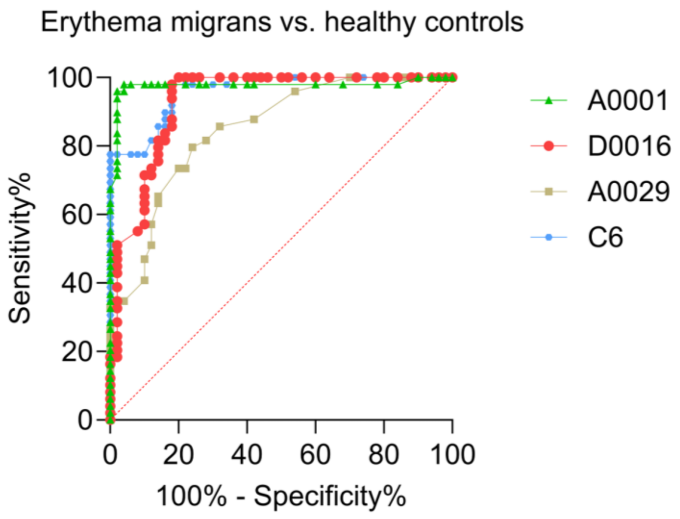

Antigens	Cut-off	AUC		95% CI		p-value
		EM	DIS	EM	DIS	EM and DIS
A0001	≥0.43	0.98	0.94-1	A0001	≥0.43	<0.0001
D0016	≥0.90	0.93	0.88-0.98	D0016	≥0.90	
A0029	≥0.99	0.85	0.78-0.92	A0029	≥0.99	
C6	≥1.1	0.96	0.93-0.99	C6	≥1.1	

Supplementary figure S5. ROC curves of A0001, D0016 and C6 in the discovery cohort. Related to Table 2. X-axis represents 100-specificity percentage and Y-axis represent sensitivity percentage of A0001, D0016 and C6. AUC, 95% CI and p-values were calculated for each antigen using GraphPad Prism v10.2.0. All serum samples were tested in duplicates. EM n=42 and HC n=50. **EM**: Erythema migrans; **HC:** Healthy controls and **ROC**: Receiver operator characteristics and **AUC:** Area under the ROC curve.

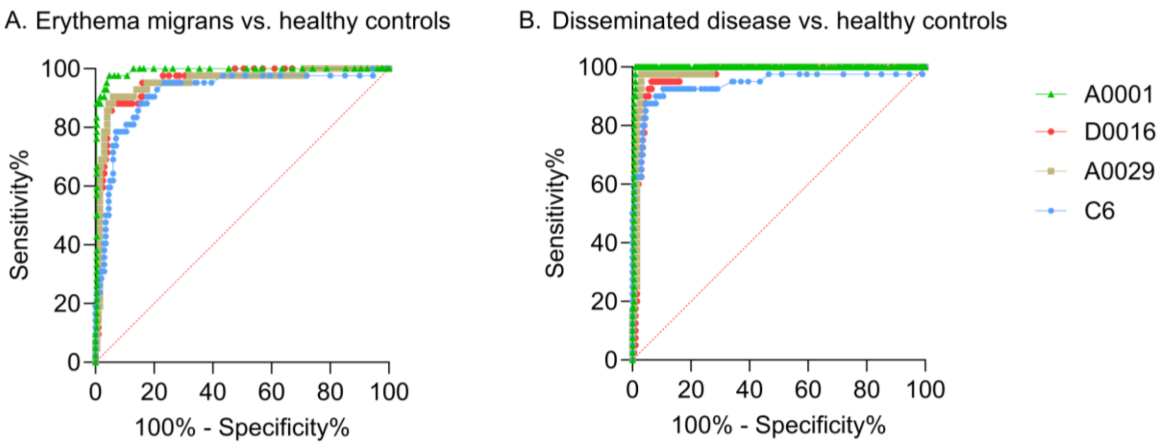

Antigens	Cut-off	AUC		95% CI		p-value
		EM	DIS	EM	DIS	EM and DIS
A0001	≥0.43	0.99	1	0.98-1	0.99-1	
D0016	≥0.90	0.95	0.97	0.92-0.98	0.95-0.99	<0.0001
A0029	≥0.99	0.95	0.98	0.91-0.99	0.96-1	
C6	≥1.1	0.92	0.94	0.87-0.97	0.89-1	

Supplementary figure S6. ROC curves of A0001, D0016, A0029 and C6 in the validation cohort: A. Erythema migrans versus healthy control sera and B. Disseminated disease versus healthy control sera. Related to Table 3. X-axis represents 100-specificity percentage and Y-axis represent sensitivity percentage of A0001, D0016, A0029 and C6. AUC, 95% CI and p-values were calculated for each antigen for both EM and DIS sera using GraphPad Prism v10.2.0. EM n=49, DIS n=40, and HC n=200. All serum samples were tested in duplicates. **HC:** Healthy controls**; EM:** Erythema migrans**; DIS:** Disseminated disease**; ROC:** Receiver operator characteristics curve and **AUC:** Area under the ROC curve.

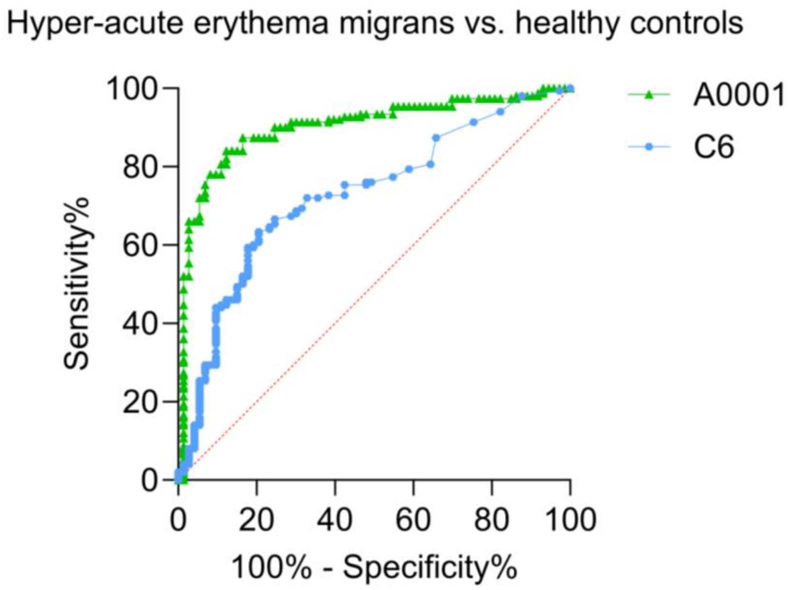

Antigens	Cut-off	AUC	95% CI	p-value
A0001	≥0.43	0.90	0.86-0.94	<0.0001
C6	≥1.1	0.72	0.65-0.79	

Supplementary figure S7. ROC curves of A0001 and C6 in acute erythema migrans sera in the prospective cohort. Related to Table 4. X-axis represents 100-specificity percentage and Y-axis represent sensitivity percentage of A0001 and C6. AUC, 95% CI and p-values were calculated for each antigen for hyper-acute EM sera using GraphPad Prism v10.2.0. EM n=150 and HC n=73. All serum samples were tested in duplicates. **HC:** Healthy controls**; EM:** Erythema migrans**; DIS:** Disseminated disease**; ROC:** Receiver operator characteristics curve and **AUC:** Area under the ROC curve.

Tables

Supplementary table S1: Tissue culture and qPCR readouts before and after treatment of murine challenged with *B. afzelii* infected ticks. Related to figure 1.

	PI				EI				LI				AT			
	**Skin**	**Bladder**	**Joint**	**Heart**	**Skin**	**Bladder**	**Joint**	**Heart**	**Skin**	**Bladder**	**Joint**	**Heart**	**Skin**	**Bladder**	**Joint**	**Heart**
Mouse 1	-	-	-	-	+	+	+	+	+	+	+	+	-	-	-	-
Mouse 2	-	-	-	-	+	+	+	+	+	+	+	+	-	-	-	-
Mouse 3	-	-	-	-	-	-	+	+	+	+	+	+	-	-	-	-
Mouse 4	-	-	-	-	+	+	+	+	+	+	+	+	-	-	-	-
Mouse 5	-	-	-	-	+	+	+	+	+	+	+	+	-	-	-	-
Mouse 6	-	-	-	-	+	+	+	+	+	+	+	+	-	-	-	-
Mouse 7	-	-	-	-	+	+	+	+	+	+	+	+	-	-	-	-
Mouse 8	-	-	-	-	+	+	+	+	+	+	+	+	-	-	-	-

Skin and bladder were harvested in MKP medium and infection (+) or no infection (-) was assessed for 6-8 weeks. DNA was extracted from joints and heart and subjected to quantitative real time PCR analysis for infection (+) or no infection (-). **PI** – Pre-immune, **EI** – Early infection, **LI** – Late infection and **AT** – Antibiotics treated (n=8; each group).

Supplementary table 2: IgM sensitivity and specificity of novel *B. afzelii* antigens in discovery cohort (determination of cut-off). Related to Table 2.

**Antigens**	**Cut-off**	**Sensitivity**			**Specificity**		
		**EM; n=49**			**HC; n=50**		
		**% (95% CI; n[α])**	**p-value (vs. C6)**	**p-value (vs. STTT)**	**% (95% CI; n[β])**	**p-value (vs. C6)**	**p-value (vs. STTT)**
A0001	≥0·22	32·7 (20-47·3; 16/49)	0·0003	ns	98 (89·4-100; 49/50)	ns	ns
D0016	≥0·40	4·0 (0·5-14; 2/49)	<0·0001	0·01	98 (89·4-100; 49/50)	ns	ns
A0029	≥0·21	32·7 (20-47·3; 16/49)	<0·0001	ns	100[¥] (89·4-100; 50/50)	ns	ns
COMBO-3	≥0·22 or 0·40 or 0·21	40·8 (27-55·8; 20/49)	0·001	ns	96 (86·3-99·5; 48/50)	ns	ns
C6*[#]	≥1·1	69·4 (54·6-81·8; 34/49)	-	-	100 (92·9-100; 50/50)	-	-
STTT[#]	-	22·5 (11·8-36·6; 11/49)	-	-	100 (92·9-100; 50)	-	-

[¥]None of the cut-off values corresponded to 98% specificity for A0029 and hence the specificity threshold was kept at 100%
*C6 Lyme index based on commercially strict cut-off value for C6; Statistical calculations were performed utilizing an exact McNemar test of paired proportions.
[#] C6 and STTT based test algorithms, evaluate both IgM and IgG responses.
[α] true positive samples
STTT: Standard two-tier testing; **EM**: Erythema migrans sera; **HC**: Healthy control sera; **ns:** non-significant

Supplementary table S3: IgG sensitivity and specificity of novel *B. afzelii* antigens in EM samples in validation cohort (validation of cut-off). Related to Table 3.

Antigens	Sensitivity						Specificity					
	EM; n=42			DIS; n=40			HC; n=200			CRC; n=70		
	% (95% CI; n^{α})	p-value (vs.C6)	p-value (vs.STTT)	% (95% CI; n^{α})	p-value (vs.C6)	p-value (vs.STTT)	% (95% CI; n^{β})	p-value (vs.C6)	p-value (vs.STTT)	% (95% CI; n^{β})	p-value (vs.C6)	p-value (vs.STTT)
D0016	50 (34·2-65·8; 21)	0·002	ns	50 (33·8-66·2; 20)	0·002	ns	98·5 (95·7-99·7; 197)	0·0002	ns	98·6 (92·3-100; 69)	0·01	ns
A0029	31 (17·6-47·0; 13)	<0·0001	ns	17·5 (7·3-32·8; 7)	<0·0001	ns	98·5 (95·7-99·7; 197)	0·0002	ns	100 (94·9-100; 70)	0·001	ns
C6*#	78·6 (63·2 -89·7; 33)	-	-	87·5 (73·2-95·8; 35)	-	-	92·5 (87·9-95·7; 185)	-	-	84·3 (75·3- 92·9;59)	-	-
STTT	40·5 (25·6-56·7; 17)	-	-	70 (53·5-83·4; 28)	-	-	98 (95-99·4; 196)	-	-	98·6 (92·3-100; 69)	-	-

*C6 Lyme index based on commercially strict cut-off value for C6; Statistical calculations were performed utilizing an exact McNemar test of paired proportions.
C6 and STTT based test algorithms, evaluate both IgM and IgG responses.
α true positive samples
β true negative samples
STTT: Standard two-tier testing; **EM**: Erythema migrans sera; **DIS**: Disseminated disease sera; **HC**: Healthy control sera; **CRC**: Cross-reactive sera and **ns:** non-significant

Supplementary table S4: IgM sensitivity and specificity of novel *B. afzelii* antigens in EM samples in validation (validation of cut-off). Related to Table 3.

Antigens	Cut-off	Sensitivity			Specificity		
		EM; n=42			HC; n=200		
		% (95% CI; n^{α})	p-value (vs. C6)	p-value (vs. STTT)	% (95% CI; n^{β})	p-value (vs. C6)	p-value (vs. STTT)
A0001	≥0·22	26·2 (13·9-42·0; 11)	<0·0001	ns	95·5 (91·6-97·9; 191)	ns	ns
D0016	≥0·40	2·4 (0·0-12·6; 1)	<0·0001	0·0001	100 (98·1-100; 200)	0·0005	ns
A0029	≥0·21	7·1 (1·5-19·5; 3)	<0·0001	0·0001	91 (86·1-94·6; 182)	ns	0·004
COMBO-3	≥0·22 or 0·40 or 0·21	26·2 (13·9-42·0; 11)	<0·0001	ns	87 (81·5-91·3; 174)	ns	0·0006
C6*#	≥1·1	78·6 (63·2-89·7; 33)	-	-	92·5 (87·9-95·7; 185)	-	-
STTT#	na	40·5 (25·7-56·7; 17)	-	-	98 (95-99·5; 196)	-	-

*C6 Lyme index based on commercially strict cut-off value for C6; Statistical calculations were performed utilizing an exact McNemar test of paired proportions.
C6 and STTT based test algorithms evaluate both IgM and IgG responses.
α true positive samples
β true negative samples
STTT: Standard two-tier testing; **EM**: Erythema migrans sera; **HC**: Healthy control sera and **ns:** non-significant
