# Supplementary material for: Diagnostic validation of novel Borrelia antigens discovered by whole-proteome microarray: Advancing early detection and test of cure for Lyme disease
Source: Cell Rep Med. 2025 May 1;6(5):102097. doi: 10.1016/j.xcrm.2025.102097 (PMC12147846; doi:10.1016/j.xcrm.2025.102097)
Supplement: Document S2. Article plus supplemental information [file mmc2.pdf]

Cell Reports
Medicine

Article

Diagnostic validation of novel *Borrelia* antigens discovered by whole-proteome microarray: Advancing early detection and test of cure for Lyme disease

Graphical abstract

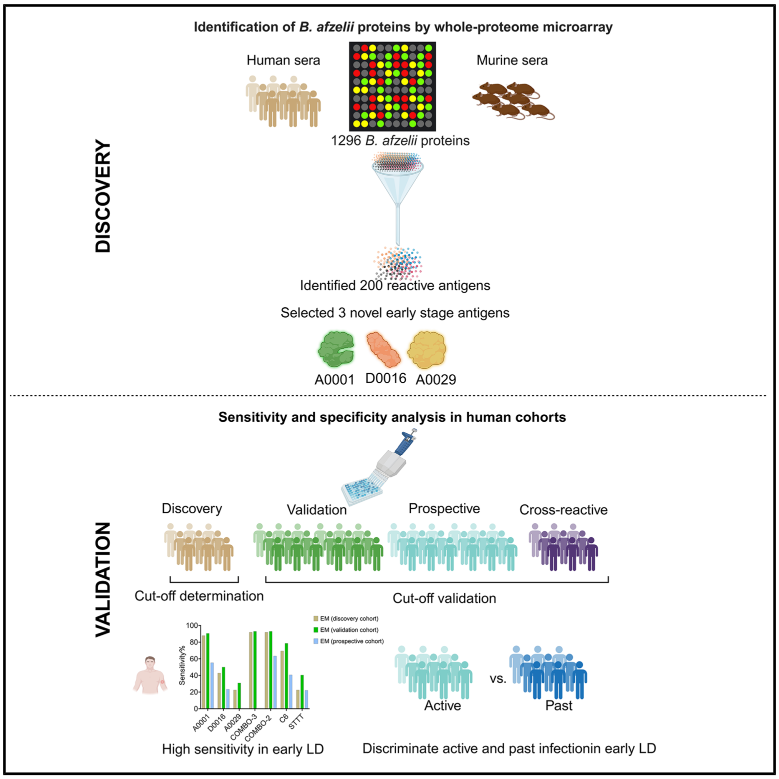

Highlights

- Whole-proteome screening of *Borrelia* identifies 3 antigens for Lyme disease diagnosis
- A0001 and COMBO-3 show improved sensitivity over conventional Lyme diagnostic tests
- A0001 and COMBO-2 detect hyper-acute erythema migrans cases with high sensitivity
- A0001 and D0016 show seroreversion, suggesting their test-of-cure potential

Authors

Abhijeet Nayak, M.E. Baarsma, Jacqueline A. van Eck, ..., Joseph J. Campo, Xiaouw Liang, Joppe W. Hovius

Correspondence

a.nayak@amsterdamumc.nl

In brief

Nayak et al. identify A0001, D0016, and A0029 as sensitive antigens for early Lyme diagnosis through whole-proteome microarray screening. They show that A0001, COMBO-2, and COMBO-3 improve early detection. A0001 and D0016 also exhibit seroreversion post-treatment, supporting their potential as test-of-cure biomarkers in early Lyme disease.

Nayak et al., 2025, Cell Reports Medicine *6*, 102097
May 20, 2025 © 2025 The Authors. Published by Elsevier Inc.
https://doi.org/10.1016/j.xcrm.2025.102097

CellPress

Cell Reports Medicine

CellPress
OPEN ACCESS

Article

Diagnostic validation of novel *Borrelia* antigens discovered by whole-proteome microarray: Advancing early detection and test of cure for Lyme disease

Abhijeet Nayak,[1,2,10,*] M.E. Baarsma,[1,2] Jacqueline A. van Eck,[1,2] Arlo Z. Randall,[3] Jeanine Ursinus,[1,2] Andy A. Teng,[3] Jozelyn V. Pablo,[3] Chris Hung,[3] Doris U.M. Wopereis,[1,2] Freek van de Schoor,[4] Calin D. Popa,[5,6] Cees C. van den Wijngaard,[7] Bart-Jan Kullberg,[4] Leo A.B. Joosten,[4,8] Herman Kuiper,[9] Joseph J. Campo,[3] Xiaouw Liang,[3] and Joppe W. Hovius[1,2]

[1]Amsterdam UMC, University of Amsterdam, Center for Infection and Molecular Medicine, Amsterdam, the Netherlands
[2]Amsterdam Institute for Immunology and Infectious Diseases, University of Amsterdam, Amsterdam, the Netherlands
[3]Antigen Discovery Inc. (ADI), ImmPORT Therapeutics Inc. dba Antigen Discovery Inc., Irvine, CA, USA
[4]Department of Internal Medicine, Radboudumc Center for Infectious Diseases and Radboud Institute of Health Sciences, Radboudumc, Nijmegen, the Netherlands
[5]Department of Rheumatology, Radboudumc, Nijmegen, the Netherlands
[6]Department of Rheumatology, Sint Maartenskliniek, Ubbergen, the Netherlands
[7]National Institute for Public Health and the Environment, Center for Infectious Disease Control, Bilthoven, the Netherlands
[8]Department of Medical Genetics, Iuliu Hatieganu University of Medicine and Pharmacy, Cluj-Napoca, Romania
[9]Amsterdam UMC, Department of Neurology, University of Amsterdam, Amsterdam, the Netherlands
[10]Lead contact
*Correspondence: a.nayak@amsterdamumc.nl
https://doi.org/10.1016/j.xcrm.2025.102097

SUMMARY

Lyme disease serodiagnosis has limited early sensitivity and cannot distinguish active from past infections. To address this, we screen a *Borrelia afzelii* whole-proteome microarray (1,296 proteins) using human (n = 149) and murine (n = 32) sera. We evaluate three early-stage antigens—BafPKo_A0001, BafPKo_D0016, and BafPKo_A0029. ELISA cutoffs are established using discovery cohort sera (n = 99) and validated with the validation (n = 242) and the prospective (n = 223) cohorts. A0001 demonstrates 87.8% sensitivity, outperforming C6 (69.4%) and STTT (22.5%) in the discovery cohort. In the validation cohort, A0001 reaches 90.5% sensitivity, surpassing C6 by 11.6% and STTT by 50%. In hyper-acute erythema migrans sera (from the prospective cohort), A0001 achieves 55.1% sensitivity, exceeding C6 and STTT by 14.6% and 33.3%, respectively. COMBO-3 and COMBO-2 yield the highest sensitivity of 92.9% and 66.1% in the validation and prospective cohort, respectively. A0001 and D0016 show enhanced and robust seroreversion after antibiotic treatment suggesting their potential as test of cure biomarkers in early Lyme disease.

INTRODUCTION

Lyme disease (LD), the most prevalent vector-borne disease in temperate regions of the Northern Hemisphere, results from infection by spirochetes of the *Borrelia burgdorferi* sensu lato complex.[1] Humans are incidental hosts and are not part of the enzootic transmission cycle of *Borrelia* but may contract the disease following the bite of an infected *Ixodes* tick. Clinically, LD presents as a spectrum of manifestations, beginning with early localized infection typically characterized by erythema migrans (EM), and progressing to disseminated stages, such as Lyme neuroborreliosis (LNB), Lyme arthritis, Lyme carditis, and acrodermatitis chronica atrophicans. In rare cases, additional severe complications may emerge.[2]

The performance of a diagnostic test is mainly evaluated by two critical metrics: sensitivity and specificity. Sensitivity measures a test's ability to correctly identify individuals with the disease (true positives), while specificity quantifies its capacity to identify those without the disease (true negatives). High sensitivity is crucial in minimizing false negatives, thereby preventing missed diagnoses where early intervention could avert disease progression. High specificity reduces false positives, preventing misdiagnosis and unnecessary interventions. For LD, achieving high sensitivity is especially challenging, notably in early-stage infection when antibody titers are low.[3,4] Similarly, background seroprevalence and asymptomatic infections present significant challenges to achieving high specificity in current diagnostic tests.[5,6]

The standard clinical diagnosis of LD is primarily serological, detecting *Borrelia*-specific antibodies in individuals presenting with compatible signs and symptoms. However, current serodiagnostic approaches exhibit notable limitations, particularly in the detection of early infection, and cannot confirm infection clearance post-treatment. Standard two-tiered testing (STTT) and modified two-tiered testing (MTTT) are the predominant

© 2025 The Authors. Published by Elsevier Inc.
This is an open access article under the CC BY license (http://creativecommons.org/licenses/by/4.0/).

diagnostic protocols implemented in routine LD serodiagnosis. STTT comprises an initial enzyme immunoassay (EIA) to optimize sensitivity, followed by a confirmatory western blot to enhance specificity.[7] However, STTT sensitivity is limited, reaching as low as 14% in cases presenting solely with EM[8] and increasing modestly to approximately 53% during convalescence.[9] STTT achieves high specificity due to the confirmatory western blot in the second tier, yet interpreting these results remains time intensive and subjective.[7] The MTTT approach, recently implemented in the United States (US), replaces the second-tier western blot with a secondary EIA, simplifying the workflow and improving early-stage sensitivity.[10,11] Although MTTT demonstrates superior performance in early LD for both US[10,12] and European cohorts,[13] this comes at a slight reduction in specificity.

Despite these advancements, current LD diagnostic tests depend on a limited subset of well-characterized *Borrelia* antigens, including outer surface protein C (OspC), the conserved C6 peptide derived from the variable major protein-like sequence expressed (VlsE) lipoprotein, the VlsE protein itself, and the flagellin protein.[14] This narrow antigenic repertoire restricts diagnostic sensitivity in early infection and specificity due to background seroprevalence. And few studies focus on identifying novel biomarkers.[15] Furthermore, currently used antigens do not differentiate between active and past infections, complicating the management of patients with persistent symptoms post-treatment. This limitation represents a significant gap in LD diagnostics, as mainly immunoglobulin (Ig)G, but even IgM, antibodies against *Borrelia* can persist for extended durations following infection clearance.[3,4]

To address these diagnostic limitations, we implemented a whole-proteome microarray comprising 1,296 *Borrelia afzelii* proteins to identify antigenic targets for serodiagnosis. We hypothesized that comprehensive profiling of the *Borrelia* proteome using sera from human and murine models would uncover novel antigenic targets that improve early-stage diagnostic sensitivity and enable differentiation between active and resolved infection. Accordingly, our objectives were to (1) identify *Borrelia* antigens with high sensitivity for early detection, particularly during the erythema migrans (EM) stage, and (2) discover biomarkers capable of distinguishing active infection from past exposure, thereby addressing the unmet need for a reliable test of cure in Lyme disease diagnostics.

Here, we report the identification and validation of *Borrelia* antigens and their combinations, demonstrating significantly improved diagnostic sensitivity without compromising on diagnostic specificity relative to currently recommended assays across multiple independent cohorts of human sera. Additionally, we present evidence supporting the potential of these antigens as test of cure biomarkers, marking the first indication of their capacity to distinguish active from past infections. These findings hold substantial promise for advancing the diagnosis and management of LD.

RESULTS

Comprehensive screening of *B. afzelii* proteome microarray with the discovery cohort and murine sera revealed several unique and known *Borrelia* antigens

We screened the whole-proteome microarray consisting of 1,296 *B afzelii* proteins with both human and murine sera in order to identify antigens that are highly sensitive in early LD and are able to classify a previous disease episode from an ongoing infection. After the initial screening and filtering, we identified 200 significantly reactive IgM and IgG antigens in both the datasets. In the discovery cohort, 24 IgM and 82 IgG antigens (4 antigens common for both IgM and IgG) were identified (Figure 1A). In the murine cohort, 55 IgM and 71 IgG antigens (12 common in both IgM and IgG) were identified (Figure 1A). Combining both the cohorts, 62 human- and 90 murine-specific antigens were identified (Figure 1A). Heatmaps were generated to depict IgG responses of selected antigens for human and murine cohort (Figures 1B and 1C; IgM responses for both datasets are depicted in Figures S1A and S1B). Most importantly, a high degree of overlap was observed in human and murine dataset with 48 common antigens. The identification of almost all known LD diagnostic antigens or their full-length counterparts in both human and murine cohorts corroborated our sample selection and the unbiased whole-proteome screening approach (Figures S2A and S2B).

A0001, D0016, and A0029 are three *Borrelia* antigens identified in early LD

To identify highly sensitive antigens, we focused on the antigens recognized in EM sera in the discovery cohort (Table 1) and early infection sera in murine cohort. We focused on the antigens that were IgG reactive in both the cohorts and identified three common antigens (Figure 2A). A0001 (PFam52 family protein/hypothetical protein; GenBank: AEL70620.1) and A0029 (hypothetical protein of unknown function; GenBank: AEL70648.1) are located on the lp54 plasmid of *B. afzelii* that is part of the core genome and required for the tick-mouse infectious stages of *Borrelia*.[16–18] D0016 (GenBank: AEL70386.1) was predicted to be a fibronectin-binding protein (a BBK32 homolog important for infectivity of *B. burgdorferi*[19] and previously used in serodiagnosis[20]; GenBank: AAC66134.1). A0001, D0016, and A0029 demonstrated significantly higher IgG responses in the EM sera compared to HC sera in the discovery cohort (Figure 2B). IgM responses against these antigens were non-significant with human sera in the discovery cohort (Figure S3A). In murine sera, all antigens demonstrated significantly higher IgG responses (Figure 2C), and D0016 showed significant IgM responses in early infection (Figure S3B). A sequence conservation and alignment for three antigens (A0001, D0016, and A0029) and their genospecies counterparts in *B. burgdorferi* sensu lato as well as relapsing fever *Borrelia* (exclusively for D0016; no orthologs could be identified for A0001 and A0029) were created using Clustal Omega multiple sequence alignment software from the European Molecular Biology Laboratory - European Bioinformatics Institute (EMBL-EBI)[21,22] database. The antigens demonstrated a homology of approximately 70% among different *B. burgdorferi sensu lato* genospecies (Figures S4A–S4C).

Determination of diagnostic cutoff values for A0001, D0016, and A0029 as single antigens and in combination

We assessed A0001, D0016, and A0029 individually or as a combination (A0001 or D0016 or A0029 referred to as COMBO-3) and compared them to C6 and STTT using the

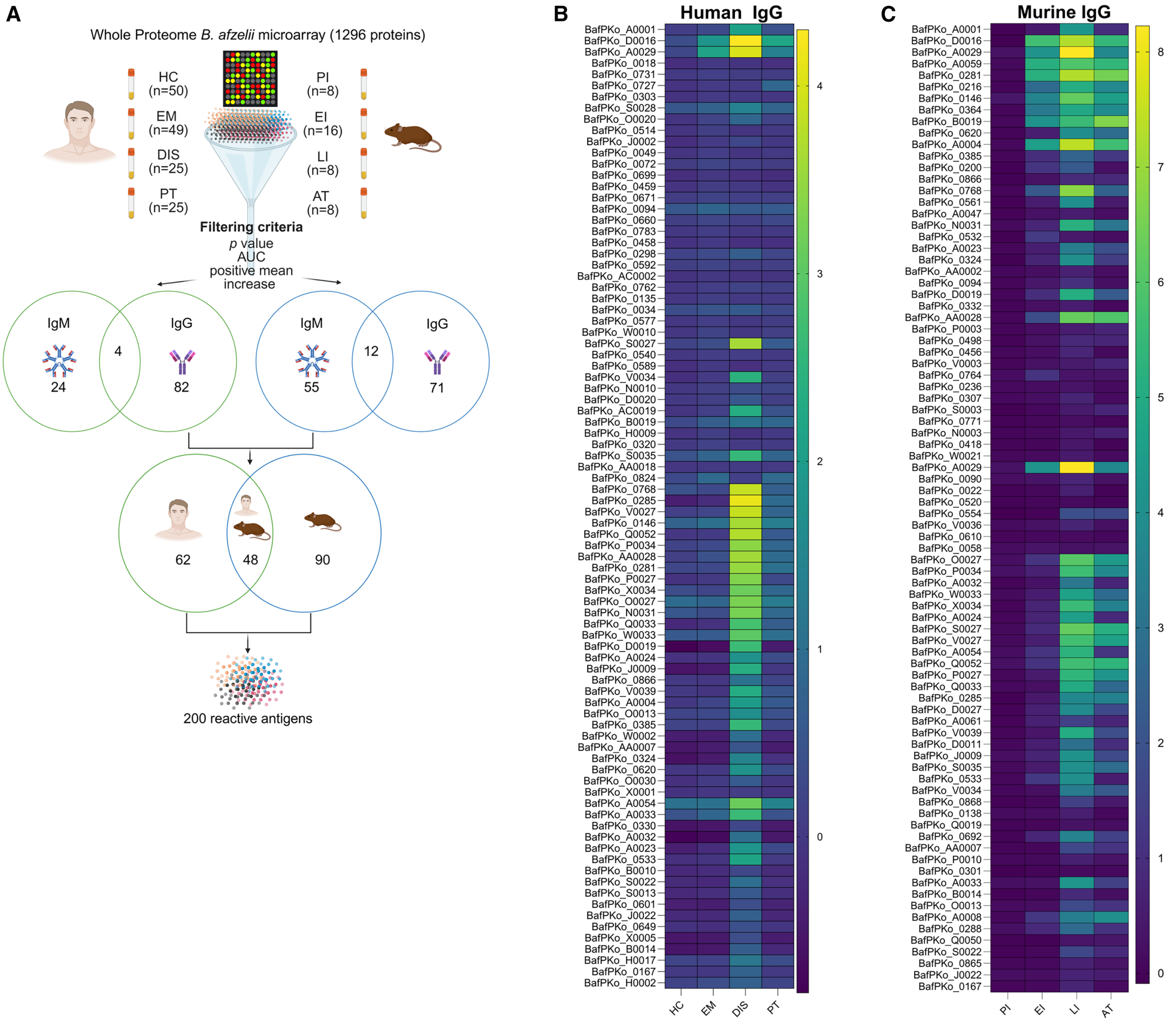

Figure 1. Screening of *B. afzelii* whole-proteome microarray with the discovery cohort and murine cohort reveals unique and known *Borrelia* antigens

(A) Workflow demonstrating whole-proteome microarray screening and downstream selection of immunoreactive antigens. 1,296 *B. afzelii* proteins were printed on a microarray chip and probed with human sera from the discovery cohort (HC, EM, DIS, and PT sera) and murine cohort (PI, EI, LI, and AT sera) to identify immunoreactive antigens. Selection of antigens was based on pre-defined filtering criteria representing *p* value, area under the curve, and positive mean increase in normalized signal intensities. Forty-eight common antigens and a total of 200 antigens with significant IgM and IgG responses were identified from both datasets.

(B) IgG antigens in the discovery cohort: heatmap depicting IgG reactivity of selected antigens in human sera IgG responses at different stages of disease as compared to healthy controls.

(C) IgG antigens in the murine cohort: heatmap depicting IgG selected murine antigens at different infection stages as compared to the pre-immune controls. Heatmaps represent the mean of normalized signal intensity of each antigen per group for both human and murine cohorts.

Figures were created using Biorender.com and GraphPad Prism v.10.2.0. All antigens including the positive and negative controls were spotted in duplicates on the microarray slides. HC, healthy control; EM, erythema migrans; DIS, disseminated disease; PT, post-treated; PI, pre-immune; EI, early infection; LI, late infection; AT, antibiotics-treated; and AUC, area under the receiver operator characteristic curve.

discovery cohort (excluding the disseminated disease and post-treatment sera). To develop a single-tier algorithm, we set the IgG specificity at 98% to determine the sensitivity using receiver operator characteristic curve (ROC curve plotted in Figure S5). A0001 demonstrated a sensitivity of 87.8% at a cut-off value of 0.43, significantly higher than C6 peptide (69.4% at a Lyme index value of 1.1, as recommend by the manufacturer) and STTT (22.5%) (Table 2). With cutoff values of 0.90 and 0.99,

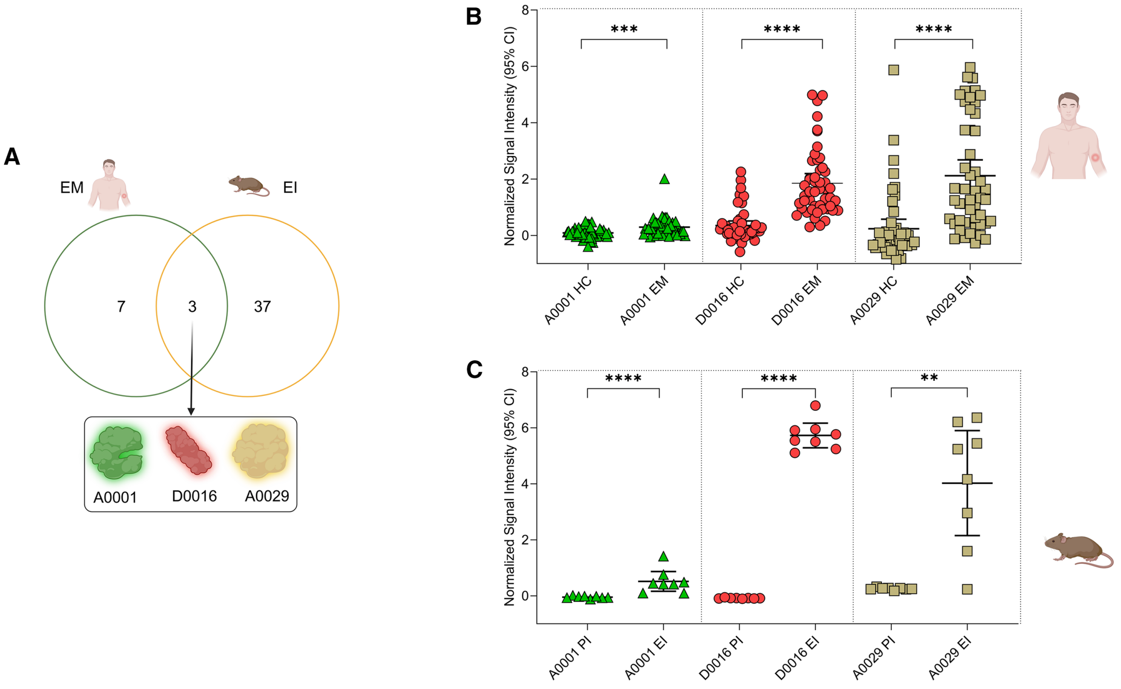

Figure 2. A0001, D0016, and A0029 are three *Borrelia* antigens identified in early Lyme disease

(A) Selection of A0001, D0016, and A0029: Venn diagram represents all antigens passing filtering criteria and A0001, D0016, and A0029 as common antigens in EM sera in humans and EI sera in mice.

(B) IgG responses of A0001, D0016, and A0029 in the discovery cohort. *x* axis represents EM sera (n = 49) and HC sera (n = 50) stage for A0001, D0016, and A0029, respectively. *y* axis represents normalized signal intensity with error bars representing 95% confidence interval (CI).

(C) IgG responses of A0001, D0016, and A0029 in the murine cohort: *x* axis represents EI sera (n = 9) and PI sera (n = 8) stage for A0001, D0016, and A0029, respectively. *y* axis represents normalized signal intensity with error bars representing 95% CI.

Figures were created using Biorender.com and GraphPad Prism v.10.2.0, and statistical significance was calculated using unpaired non-parametric Mann-Whitney test in GraphPad Prism; ****p < 0.0001, ***p < 0.001, **p < 0.01, and ns, non-significant. All antigens including the positive and negative controls were spotted in duplicates on the microarray slides. HC, healthy control; EM, erythema migrans; PI, pre-immune; and EI, early infection.

D0016 and A0029 demonstrated a sensitivity of 42.9% and 22.5%, significantly lower sensitivity than A0001 and the C6 peptide but comparable sensitivity to STTT, respectively (Table 2). For COMBO-3, the specificity—based on individual cutoff values of each antigen combined with an OR algorithm—could be maintained at 98%. COMBO-3's sensitivity was 91.8%, performing significantly better than C6 and STTT (Table 2). In our study, IgM reactivity did not significantly contribute to sensitivity of these antigens (Table S2).

A0001, COMBO-3, and COMBO-2 demonstrated superior sensitivity and specificity in two independent human LD cohorts

In an independent validation cohort (Table 1) comprising individuals with EM, disseminated disease (DIS), healthy controls (HC), and cross-reactive sera (CRC), IgG cutoffs were evaluated for A0001 and COMBO-3 and compared to C6 and STTT. In EM sera, A0001 exhibited a significantly higher sensitivity of 90.5% compared to C6 (78.6%) and STTT (40.5%), along with a specificity of 98.5%, which was significantly higher than C6 (92.5%) and comparable to the STTT (98%) (Table 3). COMBO-3 showed a sensitivity of 92.9%, comparable to but not significantly higher than A0001, while significantly outperforming both C6 and STTT. Its specificity was 96.5%, higher than C6 and similar to the STTT (Table 3).

In sera from individuals with disseminated disease, both A0001 and COMBO-3 achieved 100% sensitivity, surpassing C6 (87.5%) and the STTT (70%) (Table 3). Additional analysis of CRC indicated that A0001 and COMBO-3 achieved specificities of 98.6%, significantly exceeding C6 and comparable to the STTT (Table 3). D0016 and A0029 demonstrated lower sensitivities in EM and disseminated disease sera but achieved similar specificities in healthy control and CRC within the validation cohort (Table S3; ROC curves for A0001, D0016, A0029, and C6 are plotted in Figure S6). Consistent with findings in the discovery cohort, IgM reactivity did not significantly enhance sensitivity and instead led to lower specificity in the validation cohort (Table S4). A0029 was excluded from further analysis, both as an individual marker and in combinations, due to its low sensitivity and specificity performance within the validation cohort.

Next, we assessed antigen sensitivity and specificity in early LD using samples from a prospective cohort containing hyperacute EM sera and independent healthy control sera. In this cohort (median EM duration of 10 days), we evaluated A0001 individually and in combination with D0016 (referred to as COMBO-2). A0001 achieved a sensitivity of 55.3%, significantly

Table 1. Cohort characteristics

Cohorts	*n*	Age range (median)	F% (*n*)	Control sera HC	CRC	C6 % positivity (*n*)	STTT % positivity (*n*)[c]	Lyme disease sera EM	DIS	C6 % positivity (*n*)	STTT % positivity (*n*)[c]	Post-treated sera PT	C6 % positivity (*n*)	STTT % positivity (*n*)
Discovery	149	18–79 (55)	55.6 (79/142[a])	50	–	0.0 (0)	0.0 (0)	49[a]	25	79.7 (59)	48.6 (36)	25	64.0 (16)	0.0 (0)
Validation	352	4–81 (45.5)	49.1 (172/350[b])	200	70	9.6 (26)	1.8 (5)	40	42	82.9 (68)	54.9 (45)	–	–	–
Prospective	240	18–82 (54)	57.5 (138/240)	73	–	9.6 (7)	8.2 (6)	150	17	44.3 (74)	29.3 (44)	155 and 135[d]	36.1 (56) and 22.6 (35)	28.1 (38) and 16.3 (22)

F%, percentage of females; STTT, standard two-tier testing; HC, healthy control sera; CRC, cross-reactive sera; EM, erythema migrans sera; DIS, disseminated disease sera; PT, post-treated sera.
[a]*n* = 7 paired samples in EM sera in the discovery cohort.
[b]Gender information of *n* = 2 not available.
[c]STTT is performed when C6 is scored positive; for sake of simplicity, all C6-negative samples were also calculated as STTT negative.
[d]Number of samples corresponding to t = 6 weeks and 12 weeks.

higher than C6 (40.7%) and the STTT (22%) (Table 4; ROC curves of A0001 compared to C6 depicted in Figure S7), with a specificity of 97.2%, which, though not significantly, exceeded that of C6 (90.4%) and the STTT (91.8%) (Table 4). D0016 exhibited significantly lower sensitivity than both C6 and A0001 (data not shown). COMBO-2 reached the highest sensitivity (63.3%) in this cohort, surpassing both C6 and the STTT, with comparable specificity (93.2%) (Table 4). Compared to A0001 alone, COMBO-2 provided a 7.6% increase in sensitivity (p = 0.0004), though with a 4.0% decrease in specificity (p = 0.02) (Table 4).

Enhanced IgG seroreversion to A0001 and D0016 demonstrates superior test of cure potential as compared to C6 in post-treated LD sera

To assess the potential of A0001 and D0016 as markers for test of cure in LD, we conducted an analysis of IgG serodynamics in post-treatment LD sera. This investigation utilized only samples from a prospective cohort with longitudinal collection points at baseline (0 weeks), 6 weeks, and 12 weeks following treatment (Table 1). The majority of the post-treatment samples were obtained from patients with EM (*n* = 150), with only a small subset representing disseminated disease (*n* = 17) cases. IgG reactivity was measured against A0001, D0016, and the C6 antigen, with inclusion criteria based on baseline IgG levels exceeding the established cutoff values (≥0.43 for A0001, ≥0.90 for D0016, and ≥1.1 for C6). Pairwise comparisons were performed for sera with follow-up data, defining seroreversion as a shift to seronegativity at both 6 and 12 weeks post-treatment. In addition, samples were only included if pairwise sera were available at all the time points (for A0001 *n* = 65, for D0016 *n* = 27, and for C6 *n* = 59). Our results indicate that A0001 exhibited a robust seroreversion rate, with 95.4% of baseline-reactive samples (*n* = 65) transitioning to seronegativity by 6 and 12 weeks post-treatment (61/65) (Figure 3A). D0016 showed a more moderate seroreversion, with 66.7% of baseline-reactive samples (*n* = 27) becoming seronegative by 6 weeks (18/27), and this proportion remained consistent at 12 weeks (18/27) (Figure 3B). Notably, C6 displayed significantly lower seroreversion rates, with only 22% of initially reactive samples (*n* = 59)

Table 2. Sensitivity and specificity of *B. afzelii* antigens in the discovery cohort (determination of diagnostic cutoff)

Antigens	Cutoff	Sensitivity EM; *n* = 49 % (95% CI; *n*[c])	*p* value (vs. C6)	*p* value (vs. STTT)	Specificity HC; *n* = 50 % (95% CI; *n*[d])	*p* value (vs. C6)	*p* value (vs. STTT)
A0001	≥0.43	87.8 (75.2–95.4; 43)	0.02	<0.0001	98 (89.4–100; 49)	ns	ns
D0016	≥0.90	42.9 (28.8–57.8; 21)	0.004	<0.0001	98 (89.4–100; 49)	ns	ns
A0029	≥0.99	22.5 (11.8–36.6; 11)	<0.0001	ns	98 (89.4–100; 49)	ns	ns
COMBO-3	≥0.43 or 0.90 or 0.99	91.8 (80.4–97.7; 45)	0.01	<0.0001	98 (89.4–100; 49)	ns	ns
C6[a,b]	≥1.1	69.4 (54.6–81.8; 34)	–	–	100 (92.9–100; 50)	–	–
STTT[b]	na	22.5 (11.8–36.6; 11)	–	–	100 (92.9–100; 50)	–	–

STTT, standard two-tier testing; EM, erythema migrans sera; HC, healthy control sera; and ns, non-significant.
[a]C6 Lyme index based on commercially strict cutoff value for C6; statistical calculations were performed utilizing an exact McNemar test of paired proportions.
[b]C6- and STTT-based test algorithms, evaluate both IgM and IgG responses.
[c]True-positive samples.
[d]True-negative samples.

Table 3. Sensitivity and specificity of *B. afzelii* antigens in the validation cohort (validation of diagnostic cutoff)

	Sensitivity						Specificity					
	EM; *n* = 42			DIS; *n* = 40			HC; *n* = 200			CRC; *n* = 70		
Antigens	% (95% CI; n[c])	*p* value (vs..C6)	*p* value (vs..STTT)	% (95% CI; n[c])	*p* value (vs..C6)	*p* value (vs..STTT)	% (95% CI; n[d])	*p* value (vs..C6)	*p* value (vs..STTT)	% (95% CI; n[d])	*p* value (vs..C6)	*p* value (vs..STTT)
A0001	90.5 (77.4–97.3; 38)	0.02	<0.0001	100 (91.2–100; 40)	0.02	0.0005	98.5 (95.7–99.7; 197)	0.0002	ns	98.6 (92.3–100; 69)	0.01	ns
COMBO-3	92.9 (80.5–98.5; 39)	0.01	<0.0001	100 (91.2–100; 40)	0.02	0.0005	96.5 (92.9–98.5; 193)	ns	ns	98.6 (92.3–100; 69)	0.01	ns
C6[a,b]	78.6 (63.2–89.7; 33)	–	–	87.5 (73.2–95.8; 35)	–	–	92.5 (87.9–95.7; 185)	–	–	84.3 (75.3–92.9; 59)	–	–
STTT	40.5 (25.6–56.7; 17)	–	–	70 (53.5–83.4; 28)	–	–	98 (95–99.4; 196)	–	–	98.6 (92.3–100; 69)	–	–

STTT, standard two-tier testing; EM, erythema migrans sera; DIS, disseminated disease sera; HC, healthy control sera; CRC, cross-reactive sera; and ns, non-significant.
[a]C6 Lyme index based on commercially strict cutoff value for C6; statistical calculations were performed utilizing an exact McNemar test of paired proportions.
[b]C6- and STTT-based test algorithms, evaluate both IgM and IgG responses.
[c]True-positive samples.
[d]True-negative samples.

reverting to seronegativity by the 6-week mark and maintaining this rate at 12 weeks (13/59) (Figure 3C). Collectively, these findings underscore the potential utility of A0001 and D0016 as test of cure markers, especially in early LD, with accelerated and more consistent seroreversion kinetics compared to C6, thus supporting their clinical application in monitoring treatment efficacy in early LD.

DISCUSSION

Accurate diagnosis of LD remains a significant challenge for physicians, with current serological assays exhibiting limitations in sensitivity, specificity, and the inability to distinguish active from past infections.[4,23] In this study, we screened 1,296 *B afzelii* proteins with a whole-proteome microarray approach to identify highly sensitive early-stage and test of cure antigens. With a unique approach, we screened this array with well-characterized human sera and experimentally tick-challenged and *Borrelia*-infected murine sera and identified 200 immunoreactive *B. afzelii* antigens (with 48 common antigens). With approximately 15% of the total number of reactive antigens, our results are in line with previous studies.[15,24] We identified a plethora of immunoreactive *Borrelia* antigens, including almost all known LD diagnostic antigens used in STTT and MTTT. These findings corroborate our high-throughput approach as well as our well-characterized cohorts.

A0001, D0016, and A0029 were commonly identified in early LD in the human discovery and murine cohorts. Sequence analysis revealed A0001 to be a plasmid family 52 protein (PFam52 on lp54 plasmid) sharing 67%–70% homology to BBQ03 (GenBank: AAF07705.1), BBI42 (GenBank: AAC66192.1), and BBK53 (GenBank: AAC66137.1), the well-studied PFam52 family proteins in *B. burgdorferi*.[25] D0016 was identified as a homolog of BBK32 from *B. burgdorferi*, which was evaluated as a diagnostic antigen as well as peptides albeit with a low specificity and high cross-reactivity.[20,26] A0029, a hypothetical protein, intriguingly did not have a *B. burgdorferi* homolog, but homologs existed in *B. garinii* and *B. bavariensis*, indicating that this protein is exclusively present in European *Borrelia* genospecies.

Table 4. IgG sensitivity and specificity of *B. afzelii* antigens in the prospective cohort (validation of cutoff in hyper-acute erythema migrans sera)

	Sensitivity				Specificity			
	EM; *n* = 150				HC; *n* = 73			
Antigens	% (95% CI; n[d])	*p* value (vs..C6)	*p* value (vs. STTT)	*p* value (vs. A0001)	% (95% CI; n[e])	*p* value (vs..C6)	*p* value (vs. STTT)	*p* value (vs. A0001)
A0001	55.3 (47.0–63.5; 83)	0.002	<0.0001	–	97.3 (90.5–99.7; 71)	ns	ns	na
COMBO-2	63.3 (55.0–71.0; 95)	<0.0001	<0.0001	0.0004[b]	93.2 (84.7–97.7; 68)	ns	ns	0.02[b]
C6[a,c]	40.7 (32.7–49; 61)	–	–	–	90.4 (81.2–96.0; 66)	–	–	–
STTT[c]	22 (15.7–29.5; 33)	–	–	–	91.8 (83–96.9; 67)	–	–	–

STTT, standard two-tier testing; EM, erythema migrans sera; HC, healthy control sera; and ns, non-significant.
[a]C6 Lyme index based on commercially strict cutoff value for C6.
[b]Statistical calculations were performed utilizing an exact McNemar test of paired proportions and a one-sample t test.
[c]C6- and STTT-based test algorithms, evaluate both IgM and IgG responses.
[d]True-positive samples.
[e]True-negative samples.

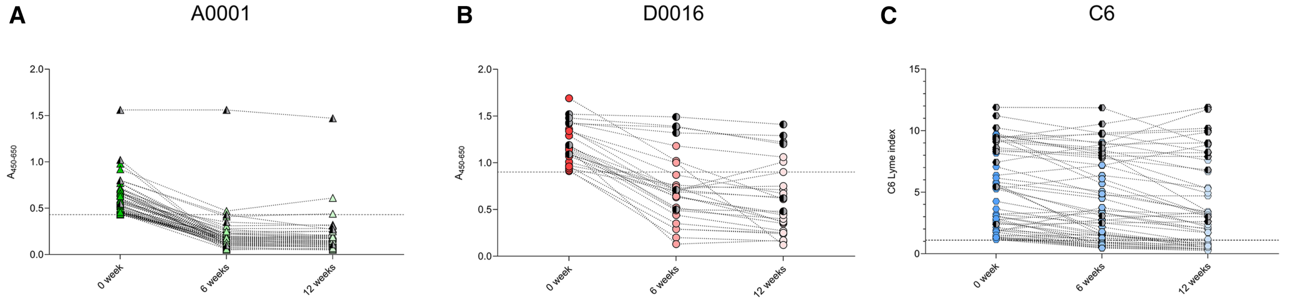

Figure 3. IgG serodynamics of A0001, D0016, and C6

IgG serodynamics were evaluated only for seropositive samples from both erythema migrans and disseminated disease sera from the prospective cohort where sequential pairwise sera were available at t = 0 weeks, 6 weeks, and 12 weeks. (A) A0001 (t = 0, 6, and 12 weeks; $n = 65$), (B) D0016 (t = 0, 6, and 12 weeks; $n = 27$), and (C) C6 (t = 0, 6, and 12 weeks; $n = 59$). Dotted lines in each graph represent cutoff values that are $\geq$0.43 for A0001, $\geq$0.90 for D0016, and commercial Lyme index of $\geq$1.1 for C6. *x* axis represents different time points of sample collection. *y* axis for A0001 and D0016 indicates absorbance 450–630 values and for C6 peptide indicates C6 Lyme index. EM sera are represented by fading-colored symbols, while disseminated disease sera are denoted by gray-black symbols for each corresponding antigen. All serum samples were tested in duplicates.

We evaluated diagnostic sensitivity and specificity in the discovery cohort with stringent specificity values (98%) with A0001 being the best performing individual antigen. COMBO-3 demonstrated the highest sensitivity of 91.8%, both significantly outperforming C6 and STTT. This indicates the diagnostic potential of these antigens in early LD. D0016 and A0029 demonstrated significantly lower sensitivity than C6, but higher or comparable to STTT. We also evaluated IgM responses and did not observe any significant increase in sensitivity, clearly indicating their limited contribution to diagnostic sensitivity and specificity. The limited contribution of IgM antibody responses to sensitivity with significantly more false positives is in line with other studies and warrants revaluation of the benefit of testing IgM responses for accurate serological diagnosis of LD.[27,28]

In the validation cohort, A0001 performed significantly better than C6 and STTT, demonstrating its potential as a highly sensitive antigen without compromising specificity, especially compared to STTT. D0016 and A0029 were lower in terms of sensitivity but on par or higher in terms of specificity. COMBO-3 again demonstrated the highest sensitivity albeit with a non-significant loss in specificity. With disseminated disease sera, A0001 demonstrated a sensitivity of 100% (and because of this, so did COMBO-3). Furthermore, A0001, D0016, and the COMBO-3 demonstrated high specificity in CRC. This suggests that either A0001 alone or COMBO-3 could be potentially implemented as a single-tier diagnostic test with an edge over existing methods of LD diagnosis.

We also tested the diagnostic accuracy in a prospective cohort. Our primary objective was to assess the diagnostic sensitivity and specificity of these antigens in hyper-acute EM sera, given that the average EM duration in the prospective cohort was significantly shorter than that in the validation cohort. Within the prospective cohort, A0001 demonstrated a sensitivity of 55.3%, which was notably higher than C6 (40.0%) and STTT (22%). COMBO-2 also exhibited excellent sensitivity (63.3%), surpassing A0001, C6, and STTT by 8%, 22.7%, and 41.3%, respectively, while maintaining high specificity. These results underscore the capacity of A0001 and COMBO-2 to detect LD with high sensitivity and specificity even at the earliest stages. This is particularly beneficial in cases where clinical diagnosis may be ambiguous, such as with nonspecific skin lesions or non-cutaneous early LD.

Moreover, in sequentially paired, treated sera from the prospective cohort, both A0001 and D0016 demonstrated a marked decrease in reactivity following antibiotic treatment in EM patients, in contrast to the more stable reactivity observed with C6. Given that EM is the most common clinical manifestation of LD, the absolute number of patients developing post-treatment Lyme disease syndrome (PTLDS) after treatment is expected to be higher than that with disseminated LD. Consequently, A0001 and D0016 could serve as potential test-of-cure antigens, supporting a reduction in unnecessary antibiotic use in PTLDS patients after early LD and promoting a shift toward symptom management. In contrast, in rare instances of active and persistent *Borrelia* infection, we hypothesize that reactivity to A0001 and D0016 may remain elevated, potentially serving as a minimally invasive indicator for considering further treatment. We recognize that this hypothesis requires validation in larger, independent cohorts and real-world clinical settings, especially for disseminated LD as these numbers were small in the prospective cohort.

C6-based assays were reasonably sensitive but not deemed specific enough as a single-tier test.[29] Importantly C6-based assays are not commercially available on the market anymore. In contrast, STTT is highly specific, but not sensitive enough, especially in early disease.[30] MTTT generally results in higher sensitivity but a slight yet undesirable loss in specificity.[31] Therefore, A0001, D0016, and COMBO-2 could be implemented as stand-alone antigens or tested in combination with or as a part of MTTT to further improve diagnostic accuracy. More recently, cell-free DNA methods for direct detection have been implemented in LD diagnosis.[32] In theory, these antigens could be supplemented in this specific test setup, resulting in a combination of direct and indirect test for LD diagnosis and further aiding in diagnostic accuracy. Our study demonstrates strong evidence that these diagnostic antigens could provide a solid basis for design and development of a robust and reliable single-tier method for LD diagnosis, improving sensitivity in early disease, while maintaining specificity and offering the ability to discriminate between a past and ongoing

Borrelia infection. Finally, with further validation across diverse cohorts and implementation on recommended diagnostic platforms, these antigens have significant potential to streamline LD diagnostics, thereby supporting more accurate treatment decisions and ultimately improving patient care.

Limitations of the study

Our study demonstrates encouraging results; however, several challenges and considerations must be acknowledged. The whole-proteome microarray utilized in this study was exclusively composed of *B. afzelii* proteins, reflecting its predominance as a clinically relevant *Borrelia* species in Europe. Consequently, we did not assess the cross-reactivity or diagnostic performance of the identified antigens against other *B. burgdorferi* sensu lato genospecies, particularly *B. burgdorferi* sensu stricto, which is the primary etiological agent of LD in the US. Moreover, the validation of these antigens using early LD sera from other endemic European regions was not conducted, which may limit the generalizability of our findings across diverse epidemiological settings. Additionally, this study did not specifically evaluate the diagnostic utility of the seven early antigens recognized in human infection. Future investigations should assess these antigens in combination with the currently discovered candidates to determine their potential for enhancing diagnostic sensitivity in clinical applications. Furthermore, the diagnostic performance of these antigens remains to be evaluated in large, well-characterized cohorts of patients with non-cutaneous early LD presentations, such as disseminated LD and LNB. Finally, the translational feasibility of integrating these antigens into a standardized, clinically recommended LD diagnostic platform is yet to be assessed.

RESOURCE AVAILABILITY

Lead contact

The resources, information, reagents, and data supporting the findings of this study are available upon reasonable request to the lead contact, Abhijeet Nayak (a.nayak@amsterdamumc.nl).

Materials availability

This study did not generate any new or unique materials. All reagents and resources used in the study are commercially available or obtained from publicly accessible sources.

Data and code availability

- Depending on the request, anonymized participant data, along with the complete study protocol (when applicable), statistical analysis plan, and/or raw data, could be made accessible for researchers for a period of 36 months following article publication to ensure proper use and interpretation. Without exceptions, data sharing will be subject to a data use agreement that aligns with institutional, ethical guidelines and participant confidentiality requirements.
- This paper does not include or report any original code.
- Any additional information required to reanalyze the data reported in this paper is available from the lead contact upon request.

ACKNOWLEDGMENTS

This work was supported by Amsterdam UMC foundation and Horsting-Stuit Foundation as part of the project "Development of a Diagnostic Test for Lyme borreliosis" under the project number 21911 awarded to J.W.H. and A.N. (extension). Amsterdam UMC foundation and Horsting-Stuit Foundation had no role in the study design; sample collection; and data collection, analysis, and interpretation nor in writing the report. The authors would like to express their sincere gratitude to Stefanie Gauw, Jasmin Ersöz, Joshua Edgar, and Adam Shandling for their excellent technical support. The authors would like to thank Wim Meijberg and Angela Yee for their support in establishing a research collaboration agreement between Amsterdam UMC and Antigen Discovery Inc.

AUTHOR CONTRIBUTIONS

A.N. and J.W.H. conceived and designed the study. A.N. and J.W.H. secured funding. A.N., M.E.B., J.A.v.E., J.U., F.v.d.S., C.D.P., C.C.v.d.W., B.-J.K., L.A. B.J., and H.K. collected samples. A.N., M.E.B., J.A.v.E., A.Z.R., J.V.P., D.U.M. W., and J.J.C. performed the experiments and data generation. A.N., M.E.B., A.Z.R., J.J.C., and X.L. performed data interpretation. A.N. wrote the manuscript, and M.E.B., J.A.v.E., J.U., J.J.C., and J.W.H. critically reviewed it. All authors approved the final version for submission.

DECLARATION OF INTERESTS

A.N., A.Z.R., and J.W.H. are inventors of a patent application describing the *Borrelia* antigens (WO2023196781A1). A.Z.R., A.A.T., J.V.P., C.H., J.J.C., and X.L. are employees of Antigen Discovery Inc., a company that commercializes proteome microarray technology. A.Z.R. was an employee of Antigen Discovery Inc. during the course of the reported study.

STAR★METHODS

Detailed methods are provided in the online version of this paper and include the following:

- KEY RESOURCES TABLE
- EXPERIMENTAL MODEL AND STUDY PARTICIPANT DETAILS
  - Murine cohort
  - Ethics
  - Human cohorts
  - Ethics
- METHOD DETAILS
  - Generation of *Borrelia afzelii* protein microarrays
  - Whole-proteome microarray screening
  - ELISA to establish diagnostic sensitivity and specificity of *Borrelia* antigens
- QUANTIFICATION AND STATISTICAL ANALYSIS

SUPPLEMENTAL INFORMATION

Supplemental information can be found online at https://doi.org/10.1016/j.xcrm.2025.102097.

Received: August 7, 2024
Revised: January 24, 2025
Accepted: April 7, 2025
Published: May 1, 2025

REFERENCES

1. Stanek, G., Wormser, G.P., Gray, J., and Strle, F. (2012). Lyme borreliosis. Lancet *379*, 461–473. https://doi.org/10.1016/S0140-6736(11)60103-7.
2. Steere, A.C., Strle, F., Wormser, G.P., Hu, L.T., Branda, J.A., Hovius, J.W. R., Li, X., and Mead, P.S. (2016). Lyme borreliosis. Nat. Rev. Dis. Primers *2*, 16090. https://doi.org/10.1038/nrdp.2016.90.
3. Branda, J.A., and Steere, A.C. (2021). Laboratory Diagnosis of Lyme Borreliosis. Clin. Microbiol. Rev. *34*, e00018-19. https://doi.org/10.1128/CMR.00018-19.
4. Marques, A.R. (2015). Laboratory diagnosis of Lyme disease: advances and challenges. Infect. Dis. Clin. *29*, 295–307. https://doi.org/10.1016/j.idc.2015.02.005.

5. Baarsma, M.E., van de Schoor, F.R., Gauw, S.A., Vrijmoeth, H.D., Ursinus, J., Goudriaan, N., Popa, C.D., Ter Hofstede, H.J., Leeflang, M.M., Kremer, K., et al. (2022). Diagnostic parameters of cellular tests for Lyme borreliosis in Europe (VICTORY study): a case-control study. Lancet Infect. Dis. *22*, 1388–1396. https://doi.org/10.1016/S1473-3099(22)00205-5.

6. van de Schoor, F.R., Baarsma, M.E., Gauw, S.A., Joosten, L.A.B., Kullberg, B.J., van den Wijngaard, C.C., and Hovius, J.W. (2019). Validation of cellular tests for Lyme borreliosis (VICTORY) study. BMC Infect. Dis. *19*, 732. https://doi.org/10.1186/s12879-019-4323-6.

7. Branda, J.A., Linskey, K., Kim, Y.A., Steere, A.C., and Ferraro, M.J. (2011). Two-tiered antibody testing for Lyme disease with use of 2 enzyme immunoassays, a whole-cell sonicate enzyme immunoassay followed by a VlsE C6 peptide enzyme immunoassay. Clin. Infect. Dis. *53*, 541–547. https://doi.org/10.1093/cid/cir464.

8. Wormser, G.P., Nowakowski, J., Nadelman, R.B., Visintainer, P., Levin, A., and Aguero-Rosenfeld, M.E. (2008). Impact of clinical variables on Borrelia burgdorferi-specific antibody seropositivity in acute-phase sera from patients in North America with culture-confirmed early Lyme disease. Clin. Vaccine Immunol. *15*, 1519–1522. https://doi.org/10.1128/CVI.00109-08.

9. Steere, A.C., McHugh, G., Damle, N., and Sikand, V.K. (2008). Prospective study of serologic tests for lyme disease. Clin. Infect. Dis. *47*, 188–195. https://doi.org/10.1086/589242.

10. Branda, J.A., Strle, K., Nigrovic, L.E., Lantos, P.M., Lepore, T.J., Damle, N. S., Ferraro, M.J., and Steere, A.C. (2017). Evaluation of Modified 2-Tiered Serodiagnostic Testing Algorithms for Early Lyme Disease. Clin. Infect. Dis. *64*, 1074–1080. https://doi.org/10.1093/cid/cix043.

11. US Food & Drug Administration (2019). In FDA clears new indications for existing Lyme disease tests that may help streamline diagnoses. https://www.fda.gov/news-events/press-announcements/fda-clears-new-indications-existing-lyme-disease-tests-may-help-streamline-diagnoses.

12. Pegalajar-Jurado, A., Schriefer, M.E., Welch, R.J., Couturier, M.R., MacKenzie, T., Clark, R.J., Ashton, L.V., Delorey, M.J., and Molins, C.R. (2018). Evaluation of Modified Two-Tiered Testing Algorithms for Lyme Disease Laboratory Diagnosis Using Well-Characterized Serum Samples. J. Clin. Microbiol. *56*, e01943-17. https://doi.org/10.1128/JCM.01943-17.

13. Baarsma, M.E., Schellekens, J., Meijer, B.C., Brandenburg, A.H., Souilljee, T., Hofhuis, A., Hovius, J.W., and van Dam, A.P. (2020). Diagnostic parameters of modified two-tier testing in European patients with early Lyme disease. Eur. J. Clin. Microbiol. Infect. Dis. *39*, 2143–2152. https://doi.org/10.1007/s10096-020-03946-0.

14. Porwancher, R.B., Hagerty, C.G., Fan, J., Landsberg, L., Johnson, B.J.B., Kopnitsky, M., Steere, A.C., Kulas, K., and Wong, S.J. (2011). Multiplex immunoassay for Lyme disease using VlsE1-IgG and pepC10-IgM antibodies: improving test performance through bioinformatics. Clin. Vaccine Immunol. *18*, 851–859. https://doi.org/10.1128/CVI.00409-10.

15. Barbour, A.G., Jasinskas, A., Kayala, M.A., Davies, D.H., Steere, A.C., Baldi, P., and Felgner, P.L. (2008). A genome-wide proteome array reveals a limited set of immunogens in natural infections of humans and white-footed mice with Borrelia burgdorferi. Infect. Immun. *76*, 3374–3389. https://doi.org/10.1128/IAI.00048-08.

16. Casjens, S., Palmer, N., van Vugt, R., Huang, W.M., Stevenson, B., Rosa, P., Lathigra, R., Sutton, G., Peterson, J., Dodson, R.J., et al. (2000). A bacterial genome in flux: the twelve linear and nine circular extrachromosomal DNAs in an infectious isolate of the Lyme disease spirochete Borrelia burgdorferi. Mol. Microbiol. *35*, 490–516. https://doi.org/10.1046/j.1365-2958.2000.01698.x.

17. Casjens, S.R., Mongodin, E.F., Qiu, W.G., Luft, B.J., Schutzer, S.E., Gilcrease, E.B., Huang, W.M., Vujadinovic, M., Aron, J.K., Vargas, L.C., et al. (2012). Genome stability of Lyme disease spirochetes: comparative genomics of Borrelia burgdorferi plasmids. PLoS One *7*, e33280. https://doi.org/10.1371/journal.pone.0033280.

18. Fraser, C.M., Casjens, S., Huang, W.M., Sutton, G.G., Clayton, R., Lathigra, R., White, O., Ketchum, K.A., Dodson, R., Hickey, E.K., et al. (1997). Genomic sequence of a Lyme disease spirochaete, Borrelia burgdorferi. Nature *390*, 580–586. https://doi.org/10.1038/37551.

19. Seshu, J., Esteve-Gassent, M.D., Labandeira-Rey, M., Kim, J.H., Trzeciakowski, J.P., Höök, M., and Skare, J.T. (2006). Inactivation of the fibronectin-binding adhesin gene bbk32 significantly attenuates the infectivity potential of Borrelia burgdorferi. Mol. Microbiol. *59*, 1591–1601. https://doi.org/10.1111/j.1365-2958.2005.05042.x.

20. Heikkila, T., Seppala, I., Saxen, H., Panelius, J., Peltomaa, M., Julin, T., Carlsson, S.A., and Lahdenne, P. (2002). Recombinant BBK32 protein in serodiagnosis of early and late Lyme borreliosis. J. Clin. Microbiol. *40*, 1174–1180. https://doi.org/10.1128/JCM.40.4.1174-1180.2002.

21. Madeira, F., Pearce, M., Tivey, A.R.N., Basutkar, P., Lee, J., Edbali, O., Madhusoodanan, N., Kolesnikov, A., and Lopez, R. (2022). Search and sequence analysis tools services from EMBL-EBI in 2022. Nucleic Acids Res. *50*, W276–W279. https://doi.org/10.1093/nar/gkac240.

22. Madeira, F., Madhusoodanan, N., Lee, J., Eusebi, A., Niewielska, A., Tivey, A.R.N., Lopez, R., and Butcher, S. (2024). The EMBL-EBI Job Dispatcher sequence analysis tools framework in 2024. Nucleic Acids Res. *52*, W521–W525. https://doi.org/10.1093/nar/gkae241.

23. Schriefer, M.E. (2015). Lyme Disease Diagnosis: Serology. Clin. Lab. Med. *35*, 797–814. https://doi.org/10.1016/j.cll.2015.08.001.

24. Xu, Y., Bruno, J.F., and Luft, B.J. (2008). Profiling the humoral immune response to Borrelia burgdorferi infection with protein microarrays. Microb. Pathog. *45*, 403–407. https://doi.org/10.1016/j.micpath.2008.09.006.

25. Groshong, A.M., Gibbons, N.E., Moore, B.P., Bellamy, W.T., and Blevins, J.S. (2024). The plasmid-encoded members of paralogous gene family 52 are dispensable to the enzootic cycle of Borrelia burgdorferi. Infect. Immun. *92*, e0021424. https://doi.org/10.1128/iai.00214-24.

26. Grazlewska, W., Holec-Gasior, L., Solowinska, K., Chmielewski, T., Fiecek, B., and Contreras, M. (2023). Epitope Mapping of BmpA and BBK32 Borrelia burgdorferi Sensu Stricto Antigens for the Design of Chimeric Proteins with Potential Diagnostic Value. ACS Infect. Dis. *9*, 2160–2172. https://doi.org/10.1021/acsinfecdis.3c00258.

27. Lager, M., Dessau, R.B., Wilhelmsson, P., Nyman, D., Jensen, G.F., Matussek, A., Lindgren, P.E., Henningsson, A.J., and ScandTick Biobank Study Group; and Baqir, H. (2019). Serological diagnostics of Lyme borreliosis: comparison of assays in twelve clinical laboratories in Northern Europe. Eur. J. Clin. Microbiol. Infect. Dis. *38*, 1933–1945. https://doi.org/10.1007/s10096-019-03631-x.

28. Joyner, G., Mavin, S., Milner, R., and Lim, C. (2022). Introduction of IgM testing for the diagnosis of acute Lyme borreliosis: a study of the benefits, limitations and costs. Eur. J. Clin. Microbiol. Infect. Dis. *41*, 671–675. https://doi.org/10.1007/s10096-021-04366-4.

29. Landry, M.L., Hassan, S., Rottmann, B.G., Pesak, S.J., Ordazzo, M., Skrzyniarz, M., Deponte, S., and Peaper, D.R. (2024). Performance of two modified two-tier algorithms for the serologic diagnosis of Lyme disease. J. Clin. Microbiol. *62*, e0013924. https://doi.org/10.1128/jcm.00139-24.

30. Leeflang, M.M.G., Ang, C.W., Berkhout, J., Bijlmer, H.A., Van Bortel, W., Brandenburg, A.H., Van Burgel, N.D., Van Dam, A.P., Dessau, R.B., Fingerle, V., et al. (2016). The diagnostic accuracy of serological tests for Lyme borreliosis in Europe: a systematic review and meta-analysis. BMC Infect. Dis. *16*, 140. https://doi.org/10.1186/s12879-016-1468-4.

31. Baarsma, M.E.E., Ursinus, J., Zaaijer, H.L., Kuiper, H., and Hovius, J.W. (2021). Diagnostic Performance of the Novel BioPlex Lyme Serological Assays in European Patients with Lyme Disease. J. Clin. Microbiol. *59*, e0320520. https://doi.org/10.1128/JCM.03205-20.

32. Branda, J.A., Lemieux, J.E., Blair, L., Ahmed, A.A., Hong, D.K., Bercovici, S., Blauwkamp, T.A., Hollemon, D., Ho, C., Strle, K., et al. (2021). Detection of Borrelia burgdorferi Cell-free DNA in Human Plasma Samples for Improved Diagnosis of Early Lyme Borreliosis. Clin. Infect. Dis. *73*, e2355–e2361. https://doi.org/10.1093/cid/ciaa858.

STAR★METHODS

KEY RESOURCES TABLE

REAGENT or RESOURCE	SOURCE	IDENTIFIER
**Antibodies**		
Goat anti-Human IgG-Fc Fragment Antibody DyLight 650 Conjugated	Bethyl Laboratories, Inc.	Cat# A80-104D5; RRID:AB_10634506
Cy3-AffiniPure F(ab')2 Fragment Goat Anti-Human IgM, Fc5µ Fragment Specific	Jackson ImmunoResearch Labs	Cat# 109-166-129; RRID:AB_2337739
Cy3-AffiniPure Goat Anti-Mouse IgG, Fc_ Fragment Specific	Jackson ImmunoResearch Labs	Cat# 115-165-071;RRID:AB_2338687
Biotin-SP-AffiniPure Goat Anti-Mouse IgM, µ Chain Specific	Jackson ImmunoResearch Labs	Cat# 115-065-075; RRID:AB_2338566
Streptavidin-conjugated SureLight P3	Columbia Biosciences Corp	Cat# D7-2212
HRP-conjugated Goat Anti-Human IgG	BioRad	Cat# 5172-2504; RRID:AB_619882
HRP-conjugated Goat Anti-Human IgM	BioRad	Cat# STAR145P; RRID:AB_1102676
HRP-conjugated Goat Anti-Mouse IgG	BioRad	Cat# 0300-0108P; RRID:AB_808614
HRP-conjugated Goat Anti-Mouse IgM	BioRad	Cat# STAR86P; RRID:AB_321866
**Bacterial and Virus Strains**		
B. afzelii strain BO23	ATCC	ATCC 51992
**Biological Samples**		
C3H/HeN mice (6–8 weeks old)	Charles River Laboratories	N/A
Murine sera (pre-immune, early infection, late infection, antibiotic-treated)	This study	17-1785-1-05
Human sera (Discovery, Validation, and Prospective Cohorts)	Amsterdam Multidisciplinary Lyme Center (AMLC), VICTORY study	N/A
Healthy control sera	Sanquin Blood Bank, general population (Netherlands)	N/A
**Chemicals, Peptides, and Recombinant Proteins**		
Recombinant *Borrelia* antigens	GenScript U.S.A./this study	Lot# U8631GA190-14/P1GC001 (BafPKo_A0001); U8631GA190-4/P1GC001 (BafPko_D0016); U8631GA190-4/P1GD002 (BafPKo_D0016); U8631GA190-9/P1GB001(BafPKo_A0029)
TMB Substrate	Sigma-Aldrich	T4444/Cas# 54827-17-7
SuperBlock™ Blocking Buffer	Thermo Fisher Scientific	Cat# 37515
**Critical Commercial Assays**		
DNeasy Blood and Tissue Kit	QIAGEN	Cat#69504
Rapid Translation System for *in vitro* protein expression	BiotechRabbit, Berlin, Germany	Cat#820710
**Deposited Data**		
Raw and processed microarray data	This paper	N/A
**Experimental Models: Organisms/Strains**		
Murine model of *Ixodes ricinus-Borrelia afzelii* infection	This study	17-1785-1-05 and DIX-271
**Oligonucleotides**		
qPCR primers for *Borrelia* detection	This study	N/A
**Recombinant DNA**		
pXT7 expression vector	This study	Antigen Discovery Inc.
**Software and Algorithms**		
GenePix 4300a microarray scanner	Molecular Devices	N/A

(*Continued on next page*)

Continued

REAGENT or RESOURCE	SOURCE	IDENTIFIER
Mapix software	Innopsys	N/A
MedCalc Statistical Software	MedCalc Software BV	Version 22.016
GraphPad Prism	GraphPad Software	Version 10.2.0
Clustal Omega	EMBL-EBI Clustal Omega < EMBL-EBI<br>Madeira et al.[21,22]<br>(manuscript main text)	N/A
M. View	EMBL-EBI Mview < EMBL-EBI<br>Madeira et al.[21,22]<br>(manuscript main text)	Version 1.67
Other		
B. afzelii proteome microarray (1296 proteins)	This study	Antigen Discovery Inc.

EXPERIMENTAL MODEL AND STUDY PARTICIPANT DETAILS

Murine cohort

Four experimental groups, each consisting of eight female C3H/HeN mice (6–8 weeks old), were obtained from Charles River Laboratories. Pre-immune serum samples were collected from all mice prior to infection to serve as baseline controls. Mice assigned to the early infection group were euthanized on day 21 post-infection (d21) to collect sera representative of early infection. From day 21 to day 25 (d21–d25), the late infection (LI) and antibiotics-treated (AT) groups received once-daily intraperitoneal injections of either 100 μL of sterile phosphate-buffered saline (PBS) or 50 mg/kg of ceftriaxone, respectively. These groups were euthanized on day 56 (d56) to obtain late infection sera and assess treatment effects. Tissue samples, including ear tissue, skin from the tick bite site, bladder, heart, and ankle joints, were collected at euthanasia. DNA was extracted from these tissues using the DNeasy Blood and Tissue Kit (QIAGEN), and quantitative PCR (qPCR) was performed to quantify *Borrelia* loads (Table S1). Additionally, bladder and skin tissues were cultured in 7 mL of MKP-II medium and monitored weekly for up to 8 weeks for the presence of viable spirochetes using dark-field microscopy.

Ethics

Murine experiments were conducted in accordance with guidelines set by the Central Commission for Animal Experiments (*Centrale Commissie Dierproeven*), the Netherlands, and European legislation. Protocols were approved by the Animal Research Ethics Committee of Amsterdam UMC (study number: 17-1785-1-05).

Human cohorts

The human sera used for screening the microarray and validating the diagnostic antigens were organized into three cohorts.

Discovery cohort (DC)

The discovery cohort was used for screening the whole-proteome microarray. It included sera from Lyme disease patients (n = 99) and healthy controls (n = 50). The Lyme disease sera were further categorized based on disease stage: early-stage erythema migrans (n = 49), disseminated/late-stage acrodermatitis chronica atrophicans (n = 25), and post-treatment sera (n = 25) from patients following antibiotic treatment. These sera were predominantly culture- and/or PCR-confirmed, with most infections attributed to *Borrelia afzelii*, as samples were exclusively collected in the Netherlands. All sera were physician-confirmed and clinically well-defined. Additionally, the erythema migrans (n = 42) and healthy control (n = 50) sera were utilized in ELISA to further evaluate the antigens and establish diagnostic cut-offs.

Validation cohort (VC)

The validation cohort was used to verify the diagnostic cut-offs of *Borrelia* antigens. This cohort included independent sera from Lyme disease patients (n = 82) and healthy controls (n = 200). The Lyme disease sera in this cohort consisted of well-characterized, physician-confirmed samples, including erythema migrans sera (n = 40) and disseminated/late-stage disease sera (n = 42), with the majority being culture- and/or PCR-confirmed (primarily infected with *B. afzelii*). Additionally, the validation cohort incorporated cross-reactive sera (n = 70) from patients with other diseases frequently considered in differential diagnoses for Lyme disease or known to commonly cross-react in serological assays. These samples allowed for further evaluation of the specificity of the diagnostic antigens.

Prospective cohort (PC)

The prospective cohort was used for to validate the diagnostic cut-offs of the antigens in hyper-acute erythema migrans (very early Lyme disease) and to evaluate the test of cure potential of the antigens in post-treatment sera. This cohort included sera from hyper-acute erythema migrans patients (n = 150), healthy controls (n = 73), and disseminated/late-stage patients (n = 17). Lyme disease

patients in this cohort, including those with hyper-acute erythema migrans and late-stage disseminated disease, were treated with antibiotics, and follow-up sera were collected at 6 weeks (t = 6; n = 155) and 12 weeks (t = 12; n = 135) post-treatment. The Lyme disease patients in this cohort were also physician-confirmed and clinically well-defined. However, nearly all disseminated cases in this cohort—unlike those in the other cohorts—were undergoing antibiotic treatment at the time of inclusion, and the majority were classified as probable rather than definite cases.

All sera used in the study had their serostatus previously determined using the standard C6 ELISA and/or the standard two-tiered testing (STTT) protocol. The Lyme disease patient sera for the discovery and validation cohorts were collected as leftover sera from the Amsterdam Multidisciplinary Lyme Center (AMLC), an academic referral center for Lyme disease in the Netherlands. These samples were collected between 1986 and 2018, as published elsewhere. In both the discovery and validation cohorts, the erythema migrans sera included both acute (0–41 days) and convalescent sera (42–83 days). The disseminated/late-stage sera consisted of late convalescent sera ($\geq$84 days). The Lyme disease sera in the prospective cohort were collected as part of the VICTORY study (2018–2020). Erythema migrans sera in the prospective cohort represented hyper-acute cases with a median erythema migrans duration of 10 days.

Healthy control sera for the discovery, validation, and prospective cohorts were sourced from the general population in the Netherlands, recruited between 2018 and 2020 as part of the VICTORY study, as well as from healthy blood donors selected by the Sanquin Blood Bank, collected between 2018 and 2019. Healthy controls were evenly distributed in terms of age, gender, and geographic location within the Netherlands. These sera were randomly assigned to the different cohorts based on their availability throughout the study duration. An overview of cohort characteristics is provided in Table 1.

Ethics

This study adhered to the Declaration of Helsinki, the Netherlands Medical Research Involving Human Subjects Act, and national and institutional guidelines. Informed consent was obtained from participants as part of the clinical studies, and selected participants consented to sample reuse for research purposes—specifically, healthy control sera in the discovery and validation cohorts, as well as Lyme disease patient sera in the prospective cohort. Leftover and de-identified Lyme disease patient sera from the discovery and validation cohorts were used in this study and, as such, fell outside the scope of the Netherlands Medical Research Involving Human Subjects Act, as indicated by the Medical Ethics Committee of Amsterdam UMC.

METHOD DETAILS

Generation of *Borrelia afzelii* protein microarrays

Proteome microarrays fabricated at Antigen Discovery, Inc. contained 1,296 *B afzelii* full-length or fragmented proteins. To construct the protein microarrays, an expressible clone library was first established using sequences from *B. afzelii* strain BO23 (ATCC 51992). Each open reading frame (ORF) sequence was amplified by PCR and inserted into the vector pXT7 by recombination in *E. coli*, creating a library of partial or complete coding DNA sequences. Each clone was quality-checked using two methods: gel electrophoresis to assess successful amplification and correct size, and Sanger sequencing verification, performed by Retrogen Inc. (San Diego, CA). Proteins were then expressed using a coupled *E. coli* cell-free *in vitro* transcription and translation (IVTT) system, the Rapid Translation System (BiotechRabbit, Berlin, Germany). The *in vitro* expressed proteins were printed onto nitrocellulose-coated glass AVID slides (Grace Bio-Labs Inc., Bend, OR), which contained nitrocellulose “pads” per slide for replicate arrays. This was done using an Omni Grid Accent robotic microarray printer (Digilabs Inc., Hopkinton, MA). Each expressed protein included a 5′ polyhistidine (His) epitope and a 3′ hemagglutinin (HA) epitope. Human and mouse immunoglobulins (IgG and IgM), as well as anti-human and anti-mouse immunoglobulins, were spotted on each subarray at multiple dilutions to control for secondary antibody reactivity and the presence of test sample immunoglobulins. A total of 32 IVTT reactions without target clones were printed onto each array as negative control spots. To ensure quality, proteome microarray printing and protein expression were verified by probing random slides with fluorescently labeled anti-His and anti-HA monoclonal antibodies. These were scanned using a GenePix 4300a microarray scanner (Molecular Devices, San Jose, CA) and quantified with Mapix software (Innopsys, Carbonne, France).

Whole-proteome microarray screening

Human and mouse sera were probed on the *B. afzelii* proteome microarrays in independent experiments. Human serum samples were diluted 1:100 in protein-free Assay Diluent (Surmodics, Inc., Eden Prairie, MN) containing 3 mg/mL DH5α *E. coli* lysate for at least 30 min before being applied to proteome microarray pads. Mouse serum samples were diluted 1:50. Sera were incubated on the microarrays overnight at 4°C on a rocker. After washing with PBS-Tween wash buffer, human serum samples were incubated with Goat anti-Human IgG-Fc Fragment Antibody DyLight 650 Conjugated (Bethyl Laboratories, Inc., Montgomery, TX, Cat#A80-104D5) and Cy3-AffiniPure F(ab')2 Fragment Goat Anti-Human IgM, Fc5μ Fragment Specific (Jackson ImmunoResearch Labs, West Grove, PA, Cat#109-166-129). Mouse serum samples were incubated with Cy3-AffiniPure Goat Anti-Mouse IgG, Fc_ Fragment Specific (Jackson ImmunoResearch Labs, Cat#115-165-071) and Biotin-SP-AffiniPure Goat Anti-Mouse IgM, μ Chain Specific (Jackson ImmunoResearch Labs, Cat#115-065-075), followed by incubation with Streptavidin-conjugated SureLight P3 (Columbia Biosciences Corp, Frederick, MD, Cat#D7-2212). Microarrays were washed and scanned using a GenePix 4300a microarray scanner. Spot signal intensities were quantified using Mapix software, then exported as.CSV files and imported into the R statistical

environment for data analysis. Local background signal was subtracted from foreground spot signals within Mapix software. Data points were floored to a value of 1 for all zero and negative values and log-transformed using the base-2 logarithm. For each sample, the median signal intensity of the IVTT negative control spots was subtracted from antigen spots, representing the log2-transformed signal-to-noise ratio. Normalized signal intensity (SI) values were interpreted as follows: an SI value of 0 indicated antibody binding equivalent to the background, an SI value of 1 indicated antibody binding twice the background, and an SI value of 2 indicated antibody binding four times the background. Since IVTT control spots captured chip, sample, and batch-level systematic effects, as well as antibody background reactivity to the *E. coli* IVTT system, this procedure normalized the data, providing a relative measure of specific antibody binding in relation to non-specific antibody binding to the IVTT controls.

ELISA to establish diagnostic sensitivity and specificity of *Borrelia* antigens

IgM and IgG responses to *E. coli*-expressed recombinant proteins (Genscript, U.S.A.) were measured using indirect ELISA. The assays were performed in a blinded manner with respect to the serostatus of all sera and all samples were tested in duplicates. Half-area 96-well ELISA microplates (Greiner Bio-One) were coated with each antigen (25 ng/25 μL per well) in 1X PBS (Fresenius Kabi). Plates were blocked with 100 μL of SuperBlock buffer (Thermo Fisher Scientific) for 1 h at room temperature, then washed with 1X PBS containing 0.05% Tween 20 (PBST), with an overflow wash volume of approximately 150 μL per well. Sera were diluted 1:250 in blocking buffer, and 25 μL of diluted serum was added to each well and incubated for 1 h at room temperature. Following another PBST wash, 25 μL of a horseradish peroxidase-conjugated (HRP) polyclonal goat anti-human or anti-mouse IgM or IgG (Bio-Rad) at a concentration of 1:10,000 was added to each well and incubated for 1 h. After washing, the plates were developed for three to 5 min, based on the reactivity of positive control sera, with 25 μL of TMB (3,3′,5,5′-tetramethylbenzidine, Sigma Aldrich). The reaction was stopped with 1M H_2SO_4, and absorbance was read at 450–650 nm using an ELISA reader (Synergy 2; BioTek).

QUANTIFICATION AND STATISTICAL ANALYSIS

Whole-proteome microarray data were quantified using Mapix software, and statistical analysis was conducted in R (version 4.1.0), as described above. All additional statistical analyses and comparisons were performed using GraphPad Prism (v10.2.0 for Windows; GraphPad Software, Boston, MA, USA) and MedCalc Statistical Software (v22.016; MedCalc Software BV, Ostend, Belgium).

Antigen filtering/selection was based on statistical significance (determined using Student's t test in R v4.1.0 with thresholds of $^{*}p < 0.05$, $^{**}p < 0 \cdot 01$ and $^{***}p < 0 \cdot 001$), a positive mean increase in normalized fluorescence signal intensity (IgM or IgG) from controls to infected samples, and area under the curve (AUC) values derived from ROC analysis in R v4.1.0. In discovery cohort, healthy controls were compared to erythema migrans and disseminated Lyme disease sera with a significance cutoff of $^{*}p < 0.05$. For the murine cohort, comparisons were made between pre-immune versus early infection sera ($^{*}p < 0.01$) and pre-immune versus late infection sera ($^{**}p < 0.001$). For AUC thresholds in the discovery cohort, IgM antigens with an AUC >0.6 (healthy controls versus erythema migrans sera) and >0.7 (healthy controls versus disseminated disease sera), were selected. For IgG responses, all antigens with an AUC of >0.7 and >0.8 was selected, respectively. In the murine cohort, antigens with an AUC of >0.7 (IgM) and AUC of >0.8 (IgG) were selected. Finally, immunoreactive antigens in both the discovery and murine cohorts were selected if a positive mean increase in normalized fluorescence signal intensity was observed. Thresolds for *p*-values and AUC were determined based on the number of IgM- and IgG-reactive antigens at different disease and infection stages in the discovery and murine cohort, respectively.

Heatmaps illustrating antigen reactivity in different groups were created using GraphPad Prism (v10.2.0 for Windows; GraphPad Software, Boston, Massachusetts, USA). Statistical significance to account for IgG responses between two groups was calculated using unpaired non-parametric Mann-Whitney test in GraphPad Prism, $^{****}p < 0 \cdot 0001$, $^{***}p < 0 \cdot 001$, $^{**}p < 0 \cdot 01$, and ns-non-significant. Receiver operating characteristic (ROC) curves and AUC values were calculated to determine cut-offs for reported *Borrelia* antigens, with 95% confidence intervals calculated using the Wilson/Brown method in GraphPad Prism (v10.2.0 for Windows; GraphPad Software, Boston, Massachusetts, USA). For sensitivity and specificity comparisons between antigens, antigen combinations, and conventional C6 and STTT methods, an Exact McNemar test was applied using MedCalc Statistical Software (v22.016; MedCalc Software BV, Ostend, Belgium). To compare individual antigens with a combined-antigen assay, a one-sample *t*-test was performed using MedCalc Statistical Software (v 22.016; MedCalc Software BV, Ostend, Belgium), as this comparison could not be conducted using the Exact McNemar test.

All statistical details including the exact *p*-values, 95% confidence intervals, test types, sample sizes (n), the definition of n (murine cohort and human cohorts comprising of serum samples), and number of replicates, are provided in corresponding figures, figure legends (main and supplemental), table headings or legends (main and supplemental), and, where applicable, in the results section.

Cell Reports Medicine, Volume *6*

Supplemental information

Diagnostic validation of novel *Borrelia* antigens discovered by whole-proteome microarray: Advancing early detection and test of cure for Lyme disease

Abhijeet Nayak, M.E. Baarsma, Jacqueline A. van Eck, Arlo Z. Randall, Jeanine Ursinus, Andy A. Teng, Jozelyn V. Pablo, Chris Hung, Doris U.M. Wopereis, Freek van de Schoor, Calin D. Popa, Cees C. van den Wijngaard, Bart-Jan Kullberg, Leo A.B. Joosten, Herman Kuiper, Joseph J. Campo, Xiaouw Liang, and Joppe W. Hovius

Supplemental data

Figures

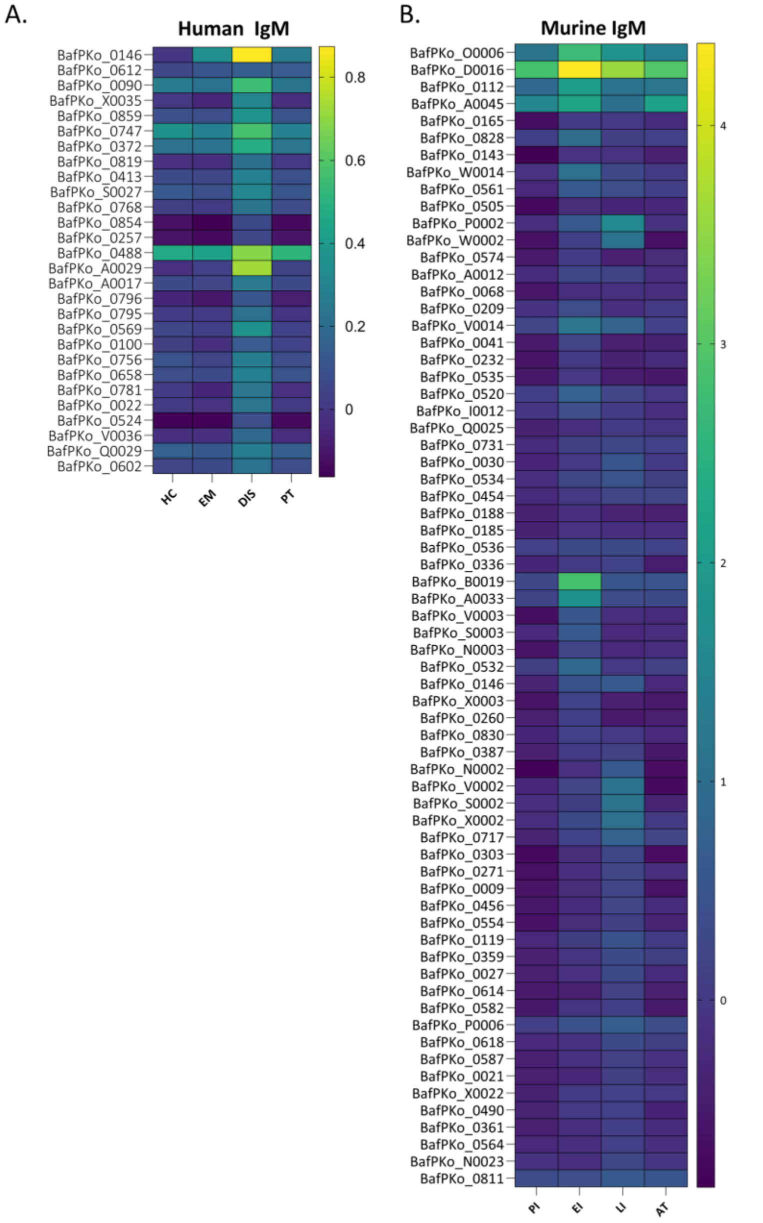

Supplementary figure S1. ***Comprehensive screening of B. afzelii proteome microarray with the discovery cohort and the murine cohort revealed several unique and known Borrelia antigens.*** Related to figure 1. 1296 *B. afzelii* proteins were printed on a microarray chip and probed with human sera from the discovery cohort (HC; n=50, EM; n=49, DIS; n=25, PT; n=25) and murine cohort (EI, PI, LI, AT; n=8) to identify immunoreactive antigens. **B. IgM antigens in the human discovery cohort**: Heat map depicting IgM reactivity of selected antigens in the discovery cohort at different stages of disease as compared to healthy controls. **C. IgM antigens in murine cohort**: Heat map depicting IgM selected murine antigens at different infection stages as compared to the pre-immune controls. Heat maps represent the mean of normalized signal intensity of each antigen per group for both human and murine cohorts. Heatmaps were created using GraphPad Prism v10.2.0. All antigens including the positive and negative controls were spotted in duplicates on the microarray slides. **DC**: Discovery cohort; **HC**: Healthy controls; **EM**: Erythema migrans; **DIS**: Disseminated disease; **PT**; Post-treated ; **PI**: Pre-immune; **EI**: Early infection; **LI**: Late infection and **AT**: Antibiotics treated.

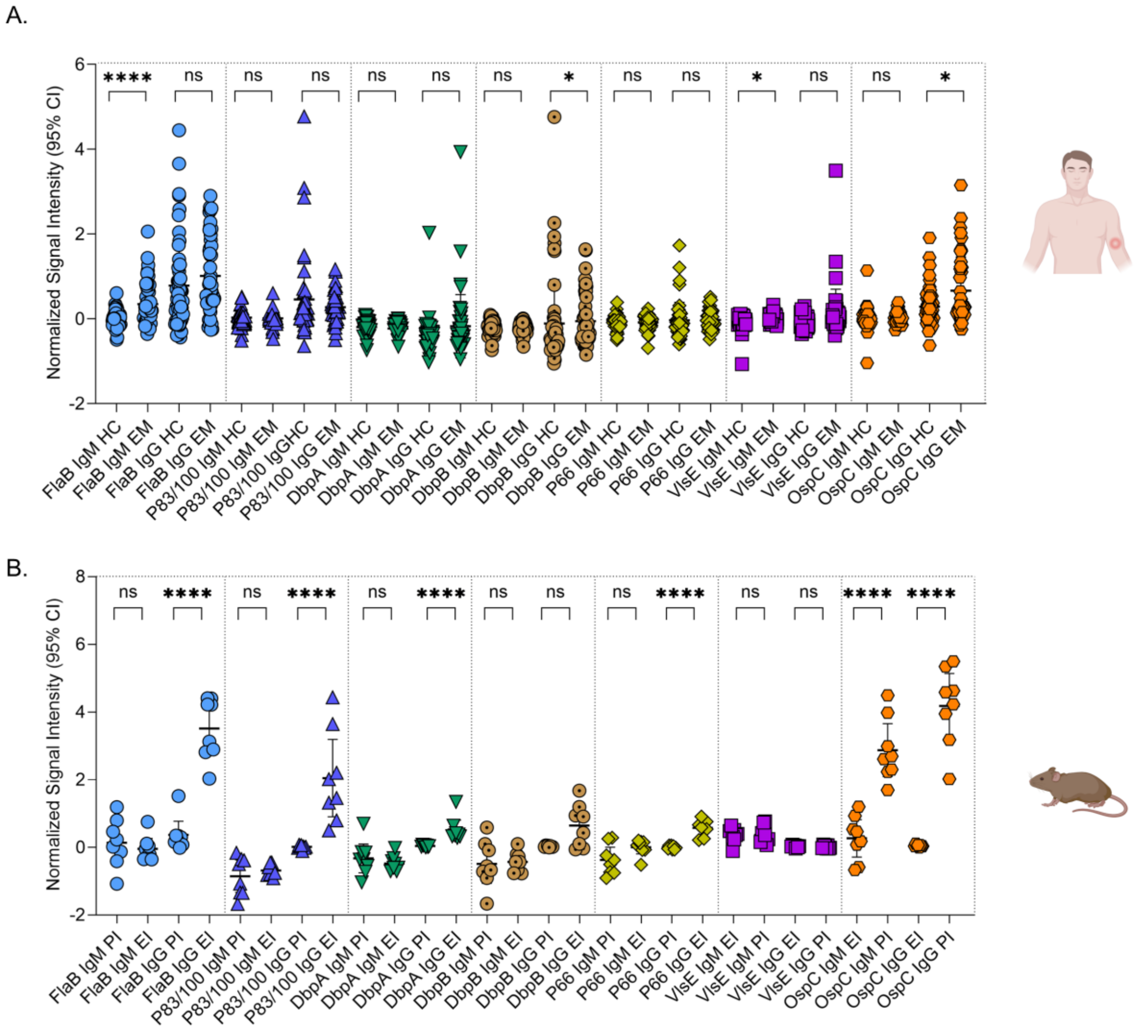

Supplementary figure S2. IgM and IgG responses of known *B. afzelii* antigens identified commonly in the discovery cohort and murine sera. Related to Figure 1. **A. IgM and IgG responses in the discovery cohort:** X-axis represents EM (n=49) and HC (n=50). Y-axis represents normalized signal intensity with error bars representing 95% CI. **C. IgM and IgG responses in murine cohort:** X-axis represents EI (n=8) and PI (n=8) stage. Y-axis represents mean normalized signal intensity with error bars representing 95% CI. Statistical significance was calculated using unpaired non-parametric Mann-Whitney test, ****p<0·0001, ***p<0·001, **p<0·01, *p<0·05, and ns- non-significant. All antigens including the positive and negative controls were spotted in duplicates on the microarray slides. Figures created using Biorender.com and GraphPad Prism v10.2.0. **HC**: Healthy controls; **EM**: Erythema migrans; **PI**: Pre-immune and **EI**: Early infection.

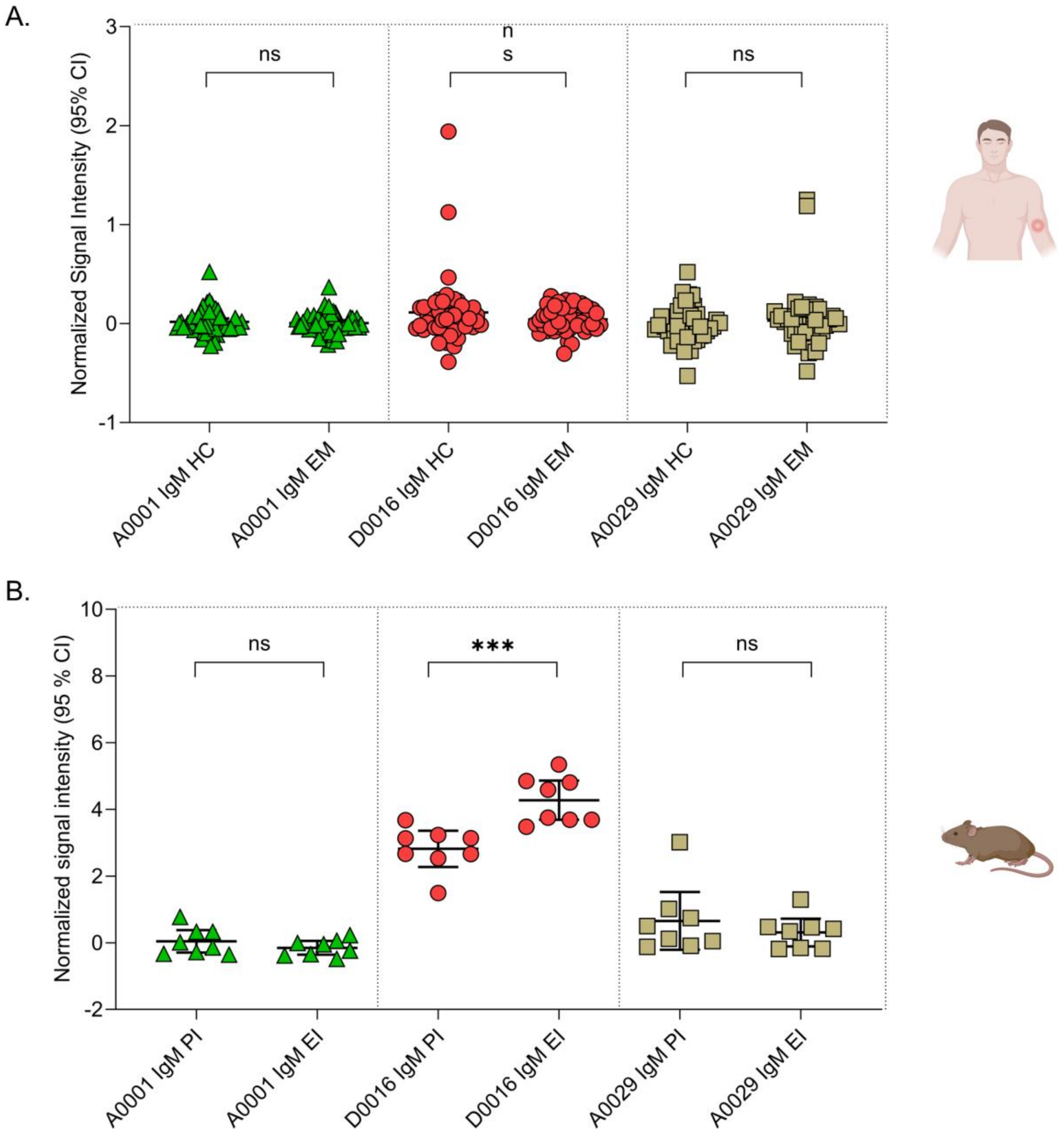

Supplementary figure S3. IgM responses. of A0001, D0016 and A0029: **A. Discovery cohort.** Related to Figure 2. X-axis represents EM sera (n=49) and HC sera (n=50) stage for A0001, D0016 and A0029, respectively. Y- axis represent normalized signal intensity with 95% CI. **B. Murine cohort:** X-axis represents EI sera (n=8) and PI sera (n=8) stage for A0001, D0016 and A0029, respectively. Y- axis represent normalized signal intensity with 95%CI. Figures were created using Biorender.com and GraphPad Prism v10.2.0 and statistical significance was calculated using unpaired non-parametric Mann-Whitney test in GraphPad Prism, ****p<0·0001, ***p<0·001, **p<0·01, and ns- non-significant. All antigens including the positive and negative controls were spotted in duplicates on the microarray slides. **HC**: Healthy controls; **EM**: Erythema migrans; **PI**: Pre-immune and **EI**: Early infection.

A. BafPKo_A0001

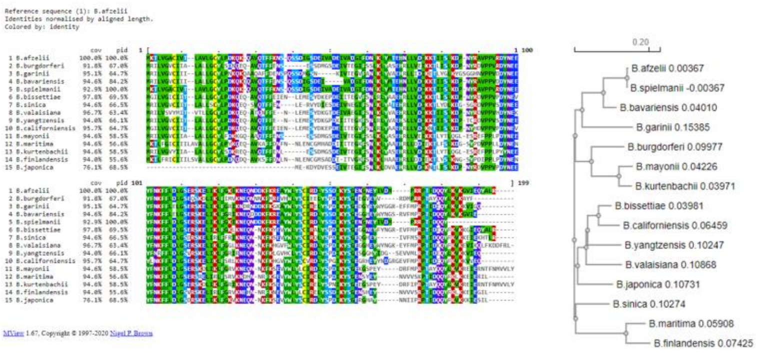

B. BafPKo_D0016

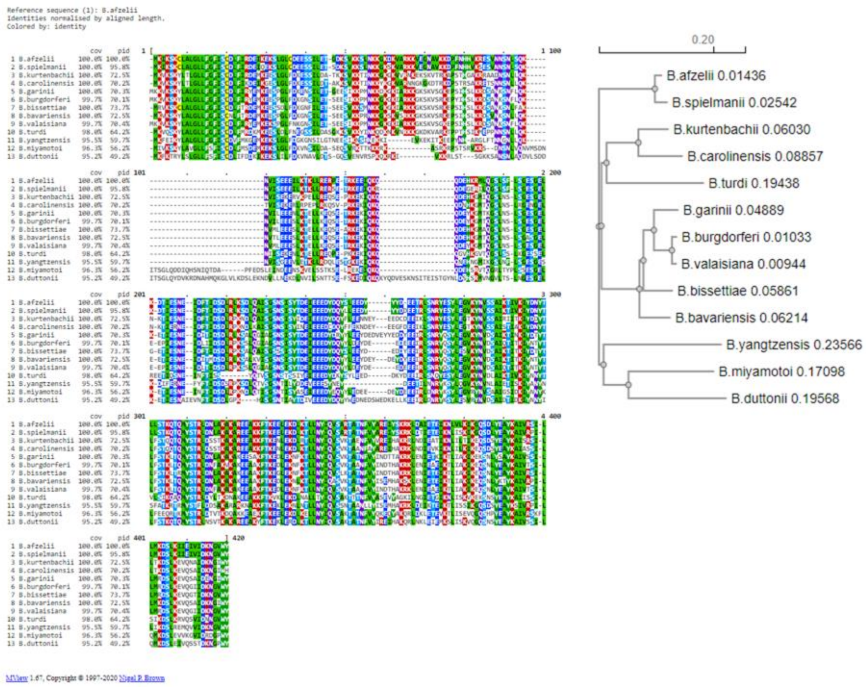

C. BafPKo_A0029

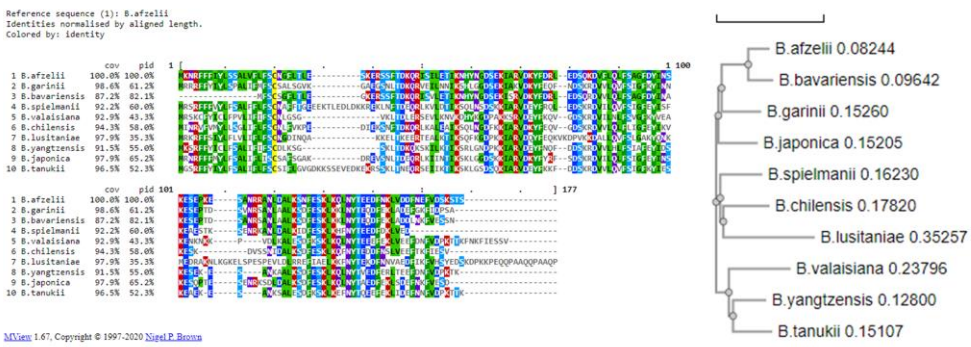

Supplementary figure S4. Sequence alignment and percent identity matrix of novel *B. afzelii* antigens. A. A0001, B. D0016 and C. A0029. Related to Figure 2. Sequence alignment files were created using Clustal Omega EMBL-EBI and alignments were created with MView Multiple sequence alignment tool EMBL-EBI. NCBI BLAST was performed, and all displayed sequences were included in the analysis. For multiple annotations from the same spirochetal genospecies only the sequence with the highest E-value was included. Phylogenetic trees were generated using Clustal Omega EMBL-EBI.

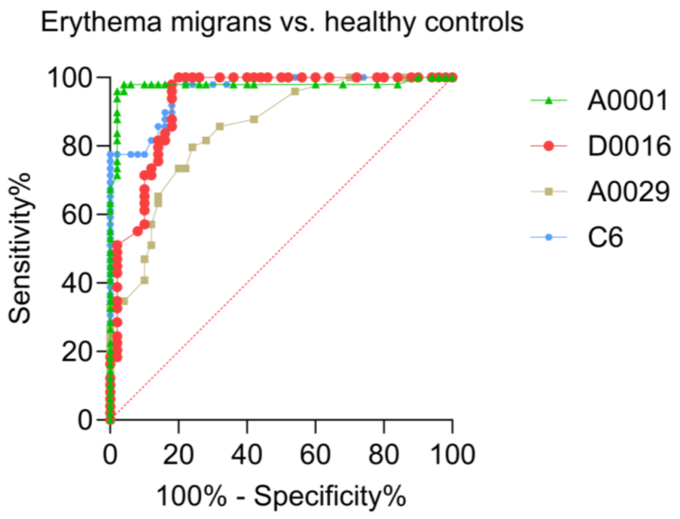

Antigens	Cut-off	AUC		95% CI		p-value
		EM	DIS	EM	DIS	EM and DIS
A0001	≥0.43	0.98	0.94-1	A0001	≥0.43	
D0016	≥0.90	0.93	0.88-0.98	D0016	≥0.90	<0.0001
A0029	≥0.99	0.85	0.78-0.92	A0029	≥0.99	
C6	≥1.1	0.96	0.93-0.99	C6	≥1.1	

Supplementary figure S5. ROC curves of A0001, D0016 and C6 in the discovery cohort. Related to Table 2. X-axis represents 100-specificity percentage and Y-axis represent sensitivity percentage of A0001, D0016 and C6. AUC, 95% CI and p-values were calculated for each antigen using GraphPad Prism v10.2.0. All serum samples were tested in duplicates. EM n=42 and HC n=50. **EM**: Erythema migrans; **HC:** Healthy controls and **ROC**: Receiver operator characteristics and **AUC:** Area under the ROC curve.

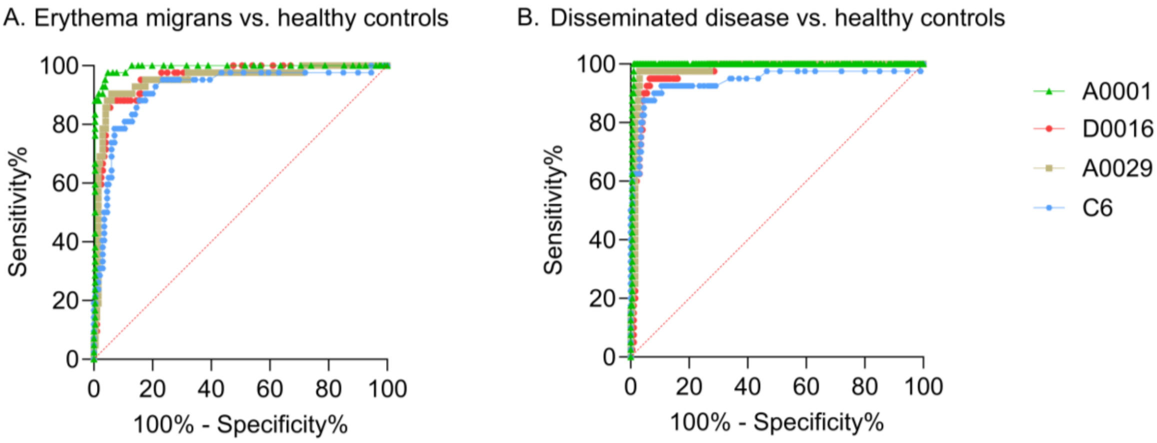

Antigens	Cut-off	AUC		95% CI		p-value
		EM	DIS	EM	DIS	EM and DIS
A0001	≥0.43	0.99	1	0.98-1	0.99-1	
D0016	≥0.90	0.95	0.97	0.92-0.98	0.95-0.99	<0.0001
A0029	≥0.99	0.95	0.98	0.91-0.99	0.96-1	
C6	≥1.1	0.92	0.94	0.87-0.97	0.89-1	

Supplementary figure S6. ROC curves of A0001, D0016, A0029 and C6 in the validation cohort: A. Erythema migrans versus healthy control sera and B. Disseminated disease versus healthy control sera. Related to Table 3. X-axis represents 100-specificity percentage and Y-axis represent sensitivity percentage of A0001, D0016, A0029 and C6. AUC, 95% CI and p-values were calculated for each antigen for both EM and DIS sera using GraphPad Prism v10.2.0. EM n=49, DIS n=40, and HC n=200. All serum samples were tested in duplicates. **HC:** Healthy controls**; EM:** Erythema migrans**; DIS:** Disseminated disease**; ROC:** Receiver operator characteristics curve and **AUC:** Area under the ROC curve.

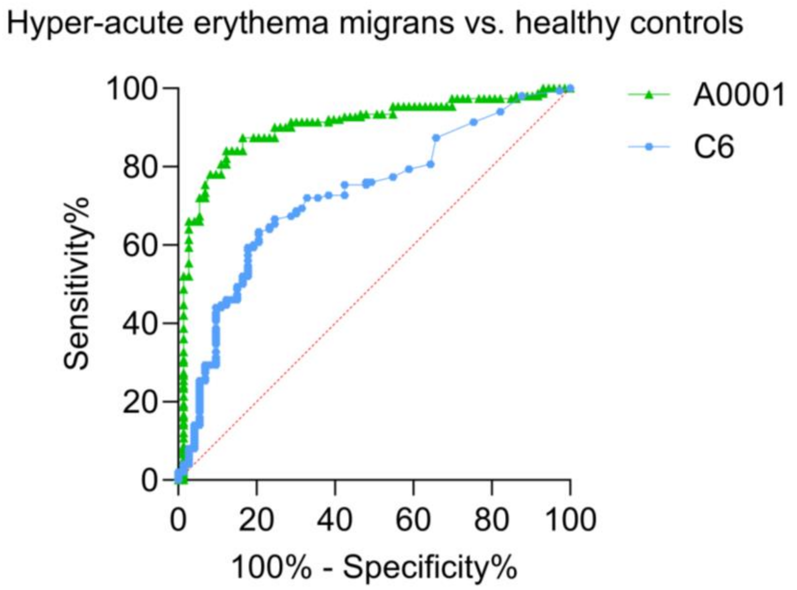

Antigens	Cut-off	AUC	95% CI	p-value
A0001	≥0.43	0.90	0.86-0.94	<0.0001
C6	≥1.1	0.72	0.65-0.79	

Supplementary figure S7. ROC curves of A0001 and C6 in acute erythema migrans sera in the prospective cohort. Related to Table 4. X-axis represents 100-specificity percentage and Y-axis represent sensitivity percentage of A0001 and C6. AUC, 95% CI and p-values were calculated for each antigen for hyper-acute EM sera using GraphPad Prism v10.2.0. EM n=150 and HC n=73. All serum samples were tested in duplicates. **HC:** Healthy controls**; EM:** Erythema migrans**; DIS:** Disseminated disease**; ROC:** Receiver operator characteristics curve and **AUC:** Area under the ROC curve.

Tables

Supplementary table S1: Tissue culture and qPCR readouts before and after treatment of murine challenged with *B. afzelii* infected ticks. Related to figure 1.

	PI				EI				LI				AT			
	**Skin**	**Bladder**	**Joint**	**Heart**	**Skin**	**Bladder**	**Joint**	**Heart**	**Skin**	**Bladder**	**Joint**	**Heart**	**Skin**	**Bladder**	**Joint**	**Heart**
Mouse 1	-	-	-	-	+	+	+	+	+	+	+	+	-	-	-	-
Mouse 2	-	-	-	-	+	+	+	+	+	+	+	+	-	-	-	-
Mouse 3	-	-	-	-	-	-	+	+	+	+	+	+	-	-	-	-
Mouse 4	-	-	-	-	+	+	+	+	+	+	+	+	-	-	-	-
Mouse 5	-	-	-	-	+	+	+	+	+	+	+	+	-	-	-	-
Mouse 6	-	-	-	-	+	+	+	+	+	+	+	+	-	-	-	-
Mouse 7	-	-	-	-	+	+	+	+	+	+	+	+	-	-	-	-
Mouse 8	-	-	-	-	+	+	+	+	+	+	+	+	-	-	-	-

Skin and bladder were harvested in MKP medium and infection (+) or no infection (-) was assessed for 6-8 weeks. DNA was extracted from joints and heart and subjected to quantitative real time PCR analysis for infection (+) or no infection (-). **PI** – Pre-immune, **EI** – Early infection, **LI** – Late infection and **AT** – Antibiotics treated (n=8; each group).

Supplementary table 2: IgM sensitivity and specificity of novel *B. afzelii* antigens in discovery cohort (determination of cut-off). Related to Table 2.

**Antigens**	**Cut-off**	**Sensitivity**			**Specificity**		
		**EM; n=49**			**HC; n=50**		
		**% (95% CI; n[α])**	**p-value (vs. C6)**	**p-value (vs. STTT)**	**% (95% CI; n[β])**	**p-value (vs. C6)**	**p-value (vs. STTT)**
A0001	≥0·22	32·7 (20-47·3; 16/49)	0·0003	ns	98 (89·4-100; 49/50)	ns	ns
D0016	≥0·40	4·0 (0·5-14; 2/49)	<0·0001	0·01	98 (89·4-100; 49/50)	ns	ns
A0029	≥0·21	32·7 (20-47·3; 16/49)	<0·0001	ns	100[¥] (89·4-100; 50/50)	ns	ns
COMBO-3	≥0·22 or 0·40 or 0·21	40·8 (27-55·8; 20/49)	0·001	ns	96 (86·3-99·5; 48/50)	ns	ns
C6*[#]	≥1·1	69·4 (54·6-81·8; 34/49)	-	-	100 (92·9-100; 50/50)	-	-
STTT[#]	-	22·5 (11·8-36·6; 11/49)	-	-	100 (92·9-100; 50)	-	-

[¥]None of the cut-off values corresponded to 98% specificity for A0029 and hence the specificity threshold was kept at 100%
*C6 Lyme index based on commercially strict cut-off value for C6; Statistical calculations were performed utilizing an exact McNemar test of paired proportions.
[#] C6 and STTT based test algorithms, evaluate both IgM and IgG responses.
[α] true positive samples
STTT: Standard two-tier testing; **EM**: Erythema migrans sera; **HC**: Healthy control sera; **ns:** non-significant

Supplementary table S3: IgG sensitivity and specificity of novel *B. afzelii* antigens in EM samples in validation cohort (validation of cut-off). Related to Table 3.

**Antigens**	**Sensitivity**						**Specificity**					
	**EM; n=42**			**DIS; n=40**			**HC; n=200**			**CRC; n=70**		
	**% (95% CI; n^{α})**	**p-value (vs.C6)**	**p-value (vs.STTT)**	**% (95% CI; n^{α})**	**p-value (vs.C6)**	**p-value (vs.STTT)**	**% (95% CI; n^{β})**	**p-value (vs.C6)**	**p-value (vs.STTT)**	**% (95% CI; n^{β})**	**p-value (vs.C6)**	**p-value (vs.STTT)**
D0016	50 (34·2-65·8; 21)	0·002	ns	50 (33·8-66·2; 20)	0·002	ns	98·5 (95·7-99·7; 197)	0·0002	ns	98·6 (92·3-100; 69)	0·01	ns
A0029	31 (17·6-47·0; 13)	<0·0001	ns	17·5 (7·3-32·8; 7)	<0·0001	ns	98·5 (95·7-99·7; 197)	0·0002	ns	100 (94·9-100; 70)	0·001	ns
C6[*#]	78·6 (63·2 -89·7; 33)	-	-	87·5 (73·2-95·8; 35)	-	-	92·5 (87·9-95·7; 185)	-	-	84·3 (75·3- 92·9;59)	-	-
STTT	40·5 (25·6-56·7; 17)	-	-	70 (53·5-83·4; 28)	-	-	98 (95-99·4; 196)	-	-	98·6 (92·3-100; 69)	-	-

[*]C6 Lyme index based on commercially strict cut-off value for C6; Statistical calculations were performed utilizing an exact McNemar test of paired proportions.
[#] C6 and STTT based test algorithms, evaluate both IgM and IgG responses.
$^{\alpha}$ true positive samples
$^{\beta}$ true negative samples
STTT: Standard two-tier testing; **EM**: Erythema migrans sera; **DIS**: Disseminated disease sera; **HC**: Healthy control sera; **CRC**: Cross-reactive sera and **ns:** non-significant

Supplementary table S4: IgM sensitivity and specificity of novel *B. afzelii* antigens in EM samples in validation (validation of cut-off). Related to Table 3.

**Antigens**	**Cut-off**	**Sensitivity**			**Specificity**		
		**EM; n=42**			**HC; n=200**		
		**% (95% CI; n^{α})**	**p-value (vs. C6)**	**p-value (vs. STTT)**	**% (95% CI; n^{β})**	**p-value (vs. C6)**	**p-value (vs. STTT)**
A0001	≥0·22	26·2 (13·9-42·0; 11)	<0·0001	ns	95·5 (91·6-97·9; 191)	ns	ns
D0016	≥0·40	2·4 (0·0-12·6; 1)	<0·0001	0·0001	100 (98·1-100; 200)	0·0005	ns
A0029	≥0·21	7·1 (1·5-19·5; 3)	<0·0001	0·0001	91 (86·1-94·6; 182)	ns	0·004
COMBO-3	≥0·22 or 0·40 or 0·21	26·2 (13·9-42·0; 11)	<0·0001	ns	87 (81·5-91·3; 174)	ns	0·0006
C6*[#]	≥1·1	78·6 (63·2-89·7; 33)	-	-	92·5 (87·9-95·7; 185)	-	-
STTT[#]	na	40·5 (25·7-56·7; 17)	-	-	98 (95-99·5; 196)	-	-

*C6 Lyme index based on commercially strict cut-off value for C6; Statistical calculations were performed utilizing an exact McNemar test of paired proportions.
[#] C6 and STTT based test algorithms evaluate both IgM and IgG responses.
$^{\alpha}$ true positive samples
$^{\beta}$ true negative samples
STTT: Standard two-tier testing; **EM**: Erythema migrans sera; **HC**: Healthy control sera and **ns:** non-significant
